# Controlling spin current polarization through non-collinear antiferromagnetism

**DOI:** 10.1038/s41467-020-17999-4

**Published:** 2020-09-16

**Authors:** T. Nan, C. X. Quintela, J. Irwin, G. Gurung, D. F. Shao, J. Gibbons, N. Campbell, K. Song, S. -Y. Choi, L. Guo, R. D. Johnson, P. Manuel, R. V. Chopdekar, I. Hallsteinsen, T. Tybell, P. J. Ryan, J. -W. Kim, Y. Choi, P. G. Radaelli, D. C. Ralph, E. Y. Tsymbal, M. S. Rzchowski, C. B. Eom

**Affiliations:** 1 Department of Materials Science and Engineering, University of Wisconsin-Madison, Madison, WI 53706 USA; 2 Department of Physics, University of Wisconsin-Madison, Madison, WI 53706 USA; 3 Department of Physics and Astronomy & Nebraska Center for Materials and Nanoscience, University of Nebraska, Lincoln, NE 68588 USA; 4 Laboratory of Atomic and Solid State Physics, Cornell University, Ithaca, NY 14853 USA; 5 Department of Materials Modeling and Characterization, KIMS, Changwon, 51508 South Korea; 6 Department of Materials Science and Engineering, POSTECH, Pohang, 37673 South Korea; 7 Clarendon Laboratory, Department of Physics, University of Oxford, Parks Road, Oxford, OX1 3PU UK; 8 ISIS Facility, Rutherford Appleton Laboratory, Chilton, Didcot, OX11 0QX UK; 9 Department of Physics and Astronomy, University College London, Gower Street, London, WC1E 6BT UK; 10 Advanced Light Source, Lawrence Berkeley National Laboratory (LBNL), 1 Cyclotron Road, Berkeley, CA 94720 USA; 11 Department of Electronic Systems, Norwegian University of Science and Technology, Trondheim, 7491 Norway; 12 Advanced Photon Source, Argonne National Laboratory, Argonne, IL 60439 USA; 13 School of Physical Sciences, Dublin City University, Dublin, 11 Ireland; 14 Kavli Institute at Cornell for Nanoscale Science, Ithaca, NY 14853 USA

**Keywords:** Spintronics, Spintronics

## Abstract

The interconversion of charge and spin currents via spin-Hall effect is essential for spintronics. Energy-efficient and deterministic switching of magnetization can be achieved when spin polarizations of these spin currents are collinear with the magnetization. However, symmetry conditions generally restrict spin polarizations to be orthogonal to both the charge and spin flows. Spin polarizations can deviate from such direction in nonmagnetic materials only when the crystalline symmetry is reduced. Here, we show control of the spin polarization direction by using a non-collinear antiferromagnet Mn_3_GaN, in which the triangular spin structure creates a low magnetic symmetry while maintaining a high crystalline symmetry. We demonstrate that epitaxial Mn_3_GaN/permalloy heterostructures can generate unconventional spin-orbit torques at room temperature corresponding to out-of-plane and Dresselhaus-like spin polarizations which are forbidden in any sample with two-fold rotational symmetry. Our results demonstrate an approach based on spin-structure design for controlling spin-orbit torque, enabling high-efficient antiferromagnetic spintronics.

## Abstract (web summary)

In the typical spin-hall effect, spin-current, charge current, and spin polarisation are all mutually perpendicular, a feature enforced by symmetry. Here, using an anti-ferromagnet with a triangular spin structure, the authors demonstrate a spin-hall effect without a perpendicular spin alignment.

## Introduction

**Fig. 1:**
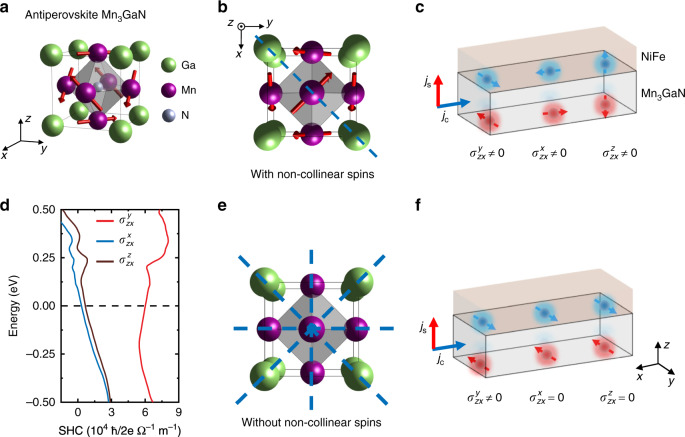
The concept of the unconventional spin-Hall effect in Mn_3_GaN. **a** The crystallographic unit cell of antiperovskite Mn_3_GaN with the antiferromagnetic Γ^5g^ spin structure where Mn spins (arrows) form a Kagome-type lattice in the (111) plane. **x**, **y**, and **z** correspond to the cubic [100], [010], and [001] axes, respectively. **b** Spin structure of Mn_3_GaN projected onto the (001) plane. The blue dashed line corresponds to the (110) mirror plane. **c** Schematic illustrations of the Py/Mn_3_GaN bilayer and the allowed spin-Hall spin polarization in the low-symmetry state (**a**). This indicates non-zero spin-Hall conductivities $\sigma _{zx}^y$, $\sigma _{zx}^x$, and $\sigma _{zx}^z$, which correspond to spin polarizations along **y**, **x**, and **z** direction, respectively (with the charge current along **x** and spin flow along **z**). **d** Calculated spin-Hall conductivities $\sigma _{zx}^y$, $\sigma _{zx}^x$, and $\sigma _{zx}^z$ for Mn_3_GaN in the antiferromagnetic phase as a function of Fermi energy. **e** Crystal structure of Mn_3_GaN without non-collinear spin structure (i.e., above the antiferromagnetic transition temperature *T*_N_) in the (001) plane, which gives rise to a high-symmetry state. **f** Allowed spin polarization in the high-symmetry state, where only the conventional spin-Hall conductivity $\sigma _{zx}^y$ is non-zero.

Current-induced spin-orbit torque enables highly efficient manipulation of magnetization for spintronic applications^1–8^. In the classical picture of current-induced magnetization dynamics^9,10^ (Fig. 1f), charge currents in a multilayer sample flowing along the in-plane direction (**x** direction) can generate out-of-plane spin currents (flowing in the **z** direction) that have spin polarization **σ**, required by symmetry to be along the **y** direction corresponding to a Rashba-like spin polarization. This particular spin current can give rise to an anti-damping spin torque in an adjacent ferromagnet, which has magnetization vector **m**, of the form **m** × (**m** × **y**). The anti-damping torque is responsible for efficient magnetization manipulation when the torque is collinear with the magnetization leading to a direct change of the effective magnetic damping. But as the anti-damping torque is restricted to lie along an in-plane direction, it is efficient for manipulating only samples with magnetic anisotropy along the in-plane **y** axis, not along the out-of-plane (**z)** direction or collinear with the current (**x** direction). Such a limitation of controlling the spin-torque or spin polarizations makes the switching of magnetic devices with magnetic anisotropy along the **x** or **z** axis nondeterministic and much less efficient (than that along **y** direction)^11^.

To efficiently and deterministically drive, for example, perpendicularly-magnetized devices that are preferred for high-density memories, an out-of-plane anti-damping torque is required. Such an out-of-plane spin-torque can originate from spin-orbit scattering from ferromagnetic interfaces^12–14^, or can arise at the interface in systems with reduced symmetry, such as in bilayers of a non-magnetic transition-metal dichalcogenide and a ferromagnetic metal^15^. However, these effects based on interface or heterostructure engineering have not been demonstrated to be strong enough for practical anti-damping switching. Here we demonstrate an alternative strategy to achieve unconventional spin-orbit torques, based on long-range non-collinear magnetic order within the *bulk* of the spin-source layer. In particular, we use the spin-Hall effect in epitaxial thin films of Mn_3_GaN, a metallic antiferromagnet that has a 120° triangular spin texture, which reduces the symmetry sufficiently to allow spin current generation with different spin polarization directions to generate unconventional spin-torques. In heterostructures of epitaxial Mn_3_GaN/permalloy, we observe not only the out-of-plane anti-damping torque, but also the anti-damping torque corresponding to a Dresselhaus-like spin polarization^16–18^, besides the conventional Rashba-like symmetry. When the non-collinear spin texture is eliminated by heating above the Néel temperature of Mn_3_GaN (345 K), the unconventional spin-torques go to zero. Such a control of the spin polarizations is coincident with our symmetry analysis and theory calculation upon the magnetic space groups across the Néel transition. Although the spin-Hall effect has been previously demonstrated in antiferromagnetic thin films, only Rashba-like symmetry has been observed^19–22^.

Mn_3_GaN is a metallic nitride with the antiperovskite crystal structure^23,24^ (identical to the perovskite structure, but with anion and cation positions interchanged) and a lattice parameter close to that of commonly used perovskite oxide substrates. In the bulk, it is known to exhibit antiferromagnetic ordering with a non-collinear Γ^5g^ Kagome-like structure (magnetic space group: ${\mathrm{R}}\bar 3m$) stabilized by the magnetic frustration of the Mn atoms in the (111) plane (Fig. 1a)^23,25^. In the (001) plane of Mn_3_GaN (Fig. 1b), the (110) plane is the only mirror plane. In this low-symmetry state, we find that charge currents along **x** generate unconventional anti-damping torque components in the form of ${\mathbf{\tau }}_x \propto {\mathbf{m}} \times ({\mathbf{m}} \times {\mathbf{x}})$ and ${\mathbf{\tau }}_z \propto {\mathbf{m}} \times ({\mathbf{m}} \times {\mathbf{z}})$ in addition to the conventional ${\mathbf{\tau }}_y \propto {\mathbf{m}} \times ({\mathbf{m}} \times {\mathbf{y}})$, which correspond to spin currents with **σ** along **x**, **z**, and **y**, respectively (Fig. 1c). These spin currents have corresponding spin-Hall conductivities $\sigma _{zx}^x$, $\sigma _{zx}^z$, and $\sigma _{zx}^y$ (in the form of $\sigma _{jk}^i$, where *i*, *j* and *k* denote the spin polarization, spin flow and charge flow directions). The symmetry allowed, and experimentally observed, non-zero spin-Hall conductivities are consistent with our linear theory calculation (Supplementary Note 9). Figure 1d shows that the $\sigma _{zx}^x$, $\sigma _{zx}^z$, and $\sigma _{zx}^y$ calculated by using the bulk Mn_3_GaN band structure are large within a wide energy window around the charge neutrality point, implying the existence of a sizable spin-Hall current even in the presence of charge carrier doping by defects. Above the antiferromagnetic-to-paramagnetic transition temperature (Néel temperature *T*_N_), disordered spins give rise to a high-symmetry state (space group: $Pm\bar 3m$) having 4 mirror planes in the crystal lattice (Fig. 1e), and consequently only the conventional spin-Hall conductivity $\sigma _{zx}^y$ can be non-zero. We list the matrices of the spin-Hall conductivity tensors, obtained from symmetry analysis and calculations, for Mn_3_GaN in antiferromagnetic and paramagnetic phases in the Supplementary Table 1.

## Results

**Fig. 2:**
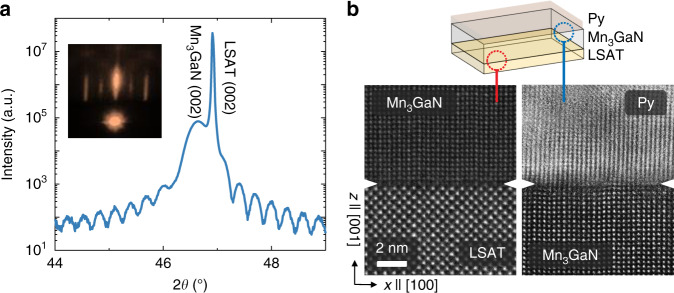
Structural characterization of the Py/Mn_3_GaN/LSAT. **a** 2*θ*-*ω* x-ray scan of the heterostructure of 10 nm Py/30 nm Mn_3_GaN on LSAT (001) substrate showing single-phase Mn_3_GaN with thickness oscillations indicating a smooth surface and sharp interface with the substrate. Inset shows reflection high-energy electron diffraction (RHEED) pattern of the specular diffraction spot for the Mn_3_GaN surface. **b** Scanning transmission electron microscope image of Py/Mn_3_GaN heterostructure on (001) LSAT substrate with the top Py/Mn_3_GaN interface (left), and the bottom Mn_3_GaN/LSAT interface (right).

Epitaxial Mn_3_GaN thin films were grown on (001) (La_0.3_Sr_0.7_)(Al_0.65_Ta_0.35_)O_3_ (LSAT) substrates by reactive magnetron sputtering with in-situ reflection high-energy electron diffraction (RHEED, see “Methods”). The out-of-plane X-ray diffraction around the (002) LSAT substrate peak shows an epitaxial Mn_3_GaN film (Fig. 2a). The distinct Kiessig fringes around the Mn_3_GaN (002) peak and the streaky RHEED pattern of the Mn_3_GaN film surface (Fig. 2a inset) indicate a high crystalline quality and a smooth film surface. We also confirmed the cube-on-cube epitaxial relationship between the Mn_3_GaN film and underlying LSAT substrate (Supplementary Note 1). Ferromagnetic permalloy Ni_81_Fe_19_ (Py) and Cu (as a spacer layer) thin films were then deposited in situ on Mn_3_GaN to form the Py/Cu/Mn_3_GaN tri-layer, and finally were patterned into device bars for spin-torque measurements. In Fig. 2b, we show the cross-sectional filtered STEM-HAADF image of a 30-nm Mn_3_GaN film on LSAT capped with 10-nm Py, which reveals sharp interfaces between both Mn_3_GaN/LSAT (left) and Py/Mn_3_GaN (right). Atomic force microscope images of the sample surface indicate an atomically-smooth surface with a surface roughness of ~0.3 nm. Using neutron diffraction, we determined that our 250-nm Mn_3_GaN films order with the bulk antiferromagnetic triangular Γ^5g^ spin structure below a Néel temperature of *T*_N_ = ~350 K (Supplementary Note 3), consistent with that for the thinner 20-nm films having *T*_N_ = 345 K, but higher than that for polycrystalline bulk samples (*T*_N_ ~ 290 K), possibly due to less grain boundaries and slight nitrogen deficiency in thin film samples (Supplementary Note 2). Using X-ray magnetic linear and circular dichroism with photoemission electron microscopy, we observe antiferromagnetic domains with size on the order of 200–300 nm (Supplementary Note 12). We note that domains with differing spin configurations can affect the unconventional spin torque, since the unconventional spin-Hall conductivity terms can be averaged out to zero under certain symmetry operations (Supplementary Note 11). The fact that we observe non-zero unconventional spin torques in Mn_3_GaN, as described below, suggests that certain antiferromagnetic domain configurations are more favorable, which is inferred to be due to a tetragonal distortion that can induce a small non-compensated magnetic moment in Mn_3_GaN thin films. This unbalanced antiferromagnetic domain population is also evidenced by the finite X-ray magnetic linear dichroism (XMLD) signal from the Mn_3_GaN films at the Mn edge for a beam area 100’s of microns in scale (Supplementary Note 12).

**Fig. 3:**
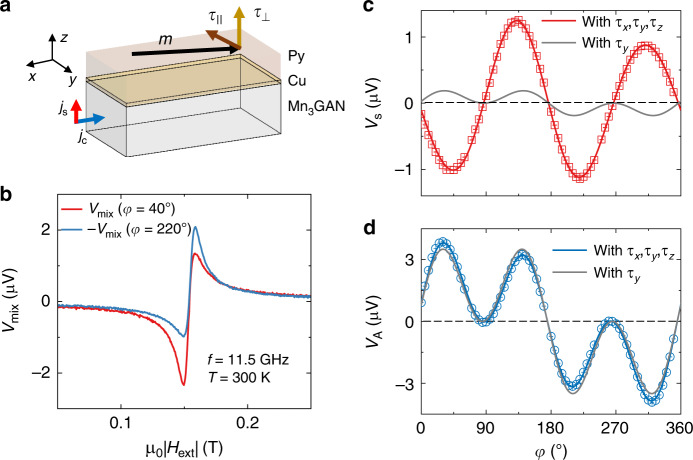
ST-FMR measurements. **a** Schematic of the ST-FMR geometry for the Py/Cu/Mn_3_GaN structures. **τ**_||_ and **τ**_**⊥**_ denote the in-plane and out-of-plane torque components, which consist of different torque terms. **b** ST-FMR spectra for the 10 nm Py/2 nm Cu/20 nm Mn_3_GaN device at 300 K (antiferromagnetic phase) with the Py magnetization oriented at 40° and 220° relative to the current axis. **c**, **d** Symmetric (**c**) and antisymmetric (**b**) ST-FMR components for the 10 nm Py/2 nm Cu/20 nm Mn_3_GaN device as a function of the in-plane magnetic field angle at 300 K. The microwave current is applied along the [100] direction (***x*** axis). The applied microwave frequency and power are 11.5 GHz and 15 dBm, respectively. The error bars indicate fitting uncertainties.

To measure the symmetry of the spin torques, we use the spin-torque ferromagnetic resonance (ST-FMR) technique (Fig. 3a)^15,26^. During the ST-FMR measurement, a microwave current applied to Mn_3_GaN produces alternating torques on the Py, and excites the Py magnetic moment into precession, generating a corresponding alternating sinusoidal change of the resistance *R* due to the anisotropic magnetoresistance (AMR) of Py. We measure a dc voltage signal *V*_mix_ across the device bar that arises from the mixing between the alternating current and changes in the device resistance. The resonance in *V*_mix_ is obtained by sweeping the external in-plane magnetic field through the Py resonance condition (see “Methods”). Both in-plane and out-of-plane torque components can then be determined individually, as the symmetric and antisymmetric part of the line shape are proportional to the amplitude of the in-plane and out-of-plane torque components, respectively. Considering only the conventional spin-Hall effect (or the Rashba–Edelstein effect and Oersted field), the in-plane and out-of-plane torque components would only have the form of **m** × (**m** × **y)** and **m** × **y**, respectively^8,27^. This corresponds to the case of samples containing materials with 2-fold rotational symmetry, in which case if **m** is inverted by rotating the in-plane magnetic field angle *φ* (with respect to **x**) by 180^°^, *V*_mix_ must retain the same amplitude but change sign, giving $V_{{\mathrm{mix}}}\left( \varphi \right) = - V_{{\mathrm{mix}}}\left( {\varphi + 180^ \circ } \right)$. Any difference in the resonance line shape between $V_{{\mathrm{mix}}}\left( \varphi \right)$ and $- V_{{\mathrm{mix}}}\left( {\varphi + 180^ \circ } \right)$ indicates the presence of an additional, unconventional torque component.

Figure 3b shows resonance spectra of a 10 nm Py/2 nm Cu/20 nm Mn_3_GaN sample with the current flow along the [100] direction for the magnetic field angle *φ* equal to 40^°^ and 220^°^, measured at room temperature when the Mn_3_GaN is in the antiferromagnetic state. The Cu insertion layer breaks the exchange coupling at the Py/Mn_3_GaN interface, but it allows the transmission of the spin current since Cu has a long spin diffusion length. We find that the *V*_mix_ (40°) and −*V*_mix_ (220°) scans are notably different in the antiferromagnetic phase, indicating the presence of unconventional torque components^15^.

To examine the torque components quantitatively, we perform ST-FMR measurements as a function of the in-plane magnetic field angle *φ*. Figure 3c and d show the angular dependence of symmetric *V*_S_ and antisymmetric *V*_A_ part for the 10 nm Py/2 nm Cu/20 nm Mn_3_GaN sample, measured at room temperature. The angular dependence of ST-FMR can be understood as the product of the AMR in Py $[dR/d\varphi \propto \sin (2\varphi )]$, with the in-plane *τ*_||_ or out-of-plane torque *τ*_⊥_ components, as $V_{\mathrm{S}} \propto \sin \left( {2\varphi } \right)\tau _\parallel$ and $V_{\mathrm{A}} \propto \sin \left( {2\varphi } \right)\tau _ \bot$. For ferromagnetic metal/normal metal bilayers (i.e., Py/Pt), the conventional anti-damping torque ${\mathbf{\tau }}_{{\mathrm{y}},{\mathrm{AD}}} \propto {\mathbf{m}} \times ({\mathbf{m}} \times {\mathbf{y}})$ and field-like torque ${\mathbf{\tau }}_{{\mathrm{y}},{\mathrm{FL}}} \propto {\mathbf{m}} \times {\mathbf{y}}$ both have a cos(*φ*) dependence, giving rise to an overall angular dependence of the form $\sin \left( {2\varphi } \right){\mathrm{cos}}(\varphi )$ for both *V*_S_ and *V*_A_. We find the angular dependence of both *V*_S_ and *V*_A_ for the Mn_3_GaN clearly deviate from this simple model (Fig. 3c and d, gray line), but can be well fitted by adding additional, unconventional torque terms with the presence of spin currents with spin polarizations oriented away from **y**. The spin currents that are polarized along **x** would generate torque [${\mathbf{\tau }}_{{\mathrm{x}},{\mathrm{AD}}} \propto {\mathbf{m}} \times ({\mathbf{m}} \times {\mathbf{x}})$ and ${\mathbf{\tau }}_{{\mathrm{x}},{\mathrm{FL}}} \propto {\mathbf{m}} \times {\mathbf{x}}$] with a sin(*φ*) dependence; while the torques with spin polarization along **z** [${\mathbf{\tau }}_{{\mathrm{z}},{\mathrm{AD}}} \propto {\mathbf{m}} \times ({\mathbf{m}} \times {\mathbf{z}})$ and ${\mathbf{\tau }}_{{\mathrm{z}},{\mathrm{FL}}} \propto {\mathbf{m}} \times {\mathbf{z}}$], since ${\mathbf{m}}$ is oriented in the plane, are independent of *φ*. We thus fit $V_{mix,\,S}(\varphi )$ and $V_{mix,\,A}(\varphi )$ to more general forms to take all possible torque terms into account:

1 $$V_{{\mathrm{mix}},\,{\mathrm{S}}}(\varphi ) \propto \sin \left( {2\varphi } \right)\left( {\tau _{{\mathrm{x}},{\mathrm{AD}}}\sin \left( \varphi \right) + \tau _{{\mathrm{y}},{\mathrm{AD}}}\cos \left( \varphi \right) + \tau _{{\mathrm{z}},{\mathrm{FL}}}} \right)$$

2 $$V_{{\mathrm{mix}},\,{\mathrm{A}}}(\varphi ) \propto \sin \left( {2\varphi } \right)\left( {\tau _{{\mathrm{x}},{\mathrm{FL}}}\sin \left( \varphi \right) + \tau _{{\mathrm{y}},{\mathrm{FL}}}\cos \left( \varphi \right) + \tau _{{\mathrm{z}},{\mathrm{AD}}}} \right)$$

We can then find non-zero anti-damping torque terms $\tau _{{\mathrm{x}},{\mathrm{AD}}}$, $\tau _{{\mathrm{y}},{\mathrm{AD}}}$, and $\tau _{{\mathrm{z}},{\mathrm{AD}}}$ (see “Methods” for calculation details) demonstrating the existence of unconventional torque originated from spin polarizations along **x** and **z**. This is consistent with the symmetry-allowed spin currents derived from the non-collinear antiferromagnetic Mn_3_GaN magnetic space group through the bulk spin-Hall effect. This mechanism is distinct from those previously reported in noncentrosymmetric systems, and in magnetic tri-layers^13,14^. The generation of the spin torque ${\mathbf{\tau }}_{{\upnu }},_{\mathrm{AD}}$ relative to the charge current density can be parameterized into the spin-torque ratio $\theta _\nu = \frac{\hbar }{{2{\mathrm{e}}}}\frac{{j_{s,\upsilon }}}{{j_c}}$, where $j_{s,\upsilon }$ is the spin current density with the spin polarization along *ν* that is absorbed by the Py, and *j*_*c*_ is the charge current density in Mn_3_GaN estimated from a parallel-conduction model. We find at room temperature that $\theta _x = - 0.013 \pm 0.0002$, $\theta _y = 0.025 \pm 0.0002$ and $\theta _z = 0.019 \pm 0.0005$, and the spin torque conductivity ($\sigma _{zx}^\nu = \frac{{\theta _\nu }}{\rho }\frac{\hbar }{{2e}}$, where *ρ* is the charge resistivity of Mn_3_GaN) to be around $\sigma _{zx}^x = - 5.9 \times 10^3\,(\hbar /2e)({\mathrm{{\Omega} }}\,m)^{ - 1}$, $\sigma _{zx}^y = 1.1 \times 10^4\,(\hbar /2e)({\mathrm{{\Omega} }}\,m)^{ - 1}$ and $\sigma _{zx}^z = 8.6 \times 10^3\,(\hbar /2e)({\mathrm{{\Omega} }}\,m)^{ - 1}$ with $\rho = 220\,\mu {\mathrm{{\Omega} }}\,{\mathrm{cm}}$. The out-of-plane field-like torque has the form ${\mathbf{\tau }}_{{\mathrm{y}},_{\mathrm{FL}}}$, dominated by the contribution from the current-induced Oersted field (see “Methods”) with no detectable ${\mathbf{\tau }}_{{\mathrm{x}},{\mathrm{FL}}}$ torque. In addition, we observe an in-plane field-like torque ${\mathbf{\tau }}_{{\mathrm{z}},{\mathrm{FL}}}$ with a large torque ratio of $\theta _{{\mathrm{FL}},{\mathrm{z}}} = - 0.15 \pm 0.0002$, which could be generated along with ${\mathbf{\tau }}_{{\mathrm{z}},{\mathrm{AD}}}$ by the spin currents polarized along **z**. The observed unconventional spin torques could also originate from spin-orbit precession^13^, in which a longitudinal spin polarized current from a ferromagnet can scatter from the ferromagnetic/normal metal interface. We note that the longitudinal spin polarized current in the non-collinear antiferromagnet and the transverse spin current that is reported in this paper could share the same physical origin^28^.

**Fig. 4:**
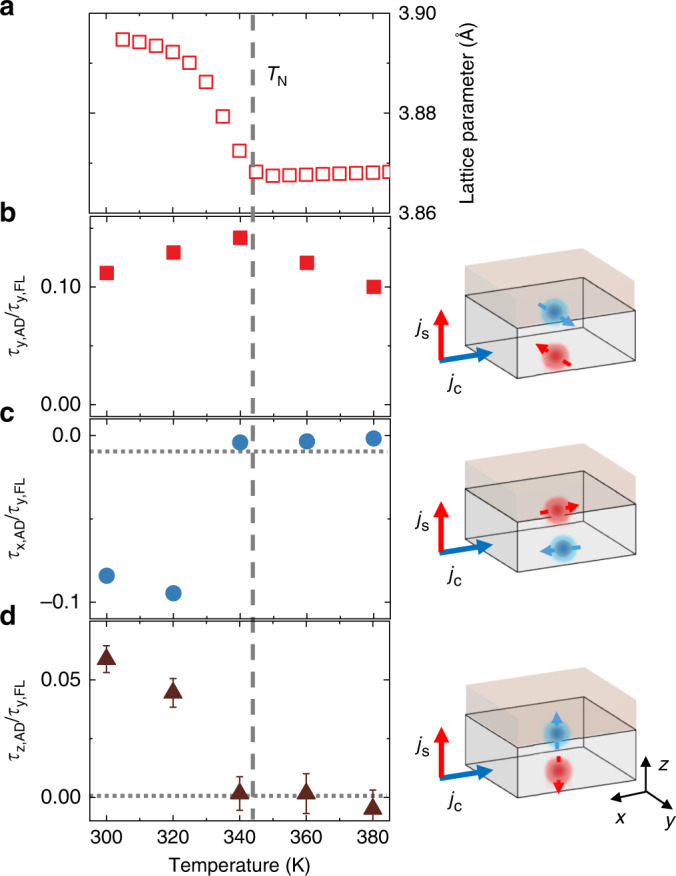
Temperature dependence of spin-orbit torques. **a** Out-of-plane lattice parameter of a 30 nm Mn_3_GaN/LSAT sample as a function of temperature; the lattice parameters anomaly indicates the Mn_3_GaN Néel temperature *T*_N_ of ~345 K. **b**–**d** The torque ratios $\tau _{{\mathrm{y}},{\mathrm{AD}}}/\tau _{{\mathrm{y}},{\mathrm{FL}}}$, $\tau _{{\mathrm{x}},{\mathrm{AD}}}/\tau _{{\mathrm{y}},{\mathrm{FL}}}$ and $\tau _{{\mathrm{z}},{\mathrm{AD}}}/\tau _{{\mathrm{y}},{\mathrm{FL}}}$ as a function of the temperature for the 10 nm Py/2 nm Cu/20 nm Mn_3_GaN device measured at the microwave frequency of 7 GHz. The schematics on the right panel show the geometry of the spin-Hall effect with different spin polarizations. The error bars indicate fitting uncertainties. Some of them are smaller than the size of the symbols.

We further confirmed the correlation between the observed unconventional spin polarization and the non-collinear spin structures in Mn_3_GaN by performing angular-dependent ST-FMR measurements across its antiferromagnetic-to-paramagnetic phase transition. The Néel temperature of the 20 nm Mn_3_GaN thin film is determined to be 345 K by tracking the temperature dependence of the out-of-plane lattice parameter (Fig. 4a) because the magnetic phase transition of Mn_3_GaN produces a region of strong negative thermal expansion^29^. Figure 4b–d show the temperature dependence (300 K–380 K) of the ratios between anti-damping torque components and the Oersted torque, $\tau _{{\mathrm{y}},{\mathrm{AD}}}/\tau _{{\mathrm{y}},{\mathrm{FL}}}$, $\tau _{{\mathrm{x}},{\mathrm{AD}}}/\tau _{{\mathrm{y}},{\mathrm{FL}}}$ and $\tau _{{\mathrm{z}},{\mathrm{AD}}}/\tau _{{\mathrm{y}},{\mathrm{FL}}}$ (extracted from the full angular-dependent ST-FMR measured at each temperature, see Supplementary Note 7). The unconventional torque ratios $\tau _{{\mathrm{x}},{\mathrm{AD}}}/\tau _{{\mathrm{y}},{\mathrm{FL}}}$ and $\tau _{{\mathrm{z}},{\mathrm{AD}}}/\tau _{{\mathrm{y}},{\mathrm{FL}}}$ vanish when the sample temperature is above the Néel temperature, while the conventional component $\tau _{{\mathrm{y}},{\mathrm{AD}}}/\tau _{{\mathrm{y}},{\mathrm{FL}}}$ remains non-zero, with a weak peak near the transition temperature (a similar peak in $\tau _{{\mathrm{y}},{\mathrm{AD}}}$ has been observed near the Curie temperature of Fe_x_Pt_1-x_ alloys^30^). The vanishing of $\tau _{{\mathrm{x}},{\mathrm{AD}}}/\tau _{{\mathrm{y}},{\mathrm{FL}}}$ and $\tau _{{\mathrm{z}},{\mathrm{AD}}}/\tau _{{\mathrm{y}},{\mathrm{FL}}}$ directly demonstrates the strong correlation between the non-collinear spin structure and the existence of the unconventional spin torques. We also find that the unconventional torques persist at temperatures well below the Néel temperature (Supplementary Note 8), but decrease gradually at lower temperature with the increase of the uncompensated moment and the onset of the anomalous Hall effect in Mn_3_GaN (Supplementary Note 4 and 5). The correlation between the observed unconventional spin-orbit torques, the uncompensated magnetic moment and the anomalous Hall effect in Mn_3_GaN at low temperatures requires further study.

## Discussion

In summary, we have demonstrated the generation of unconventional spin-orbit torque based on low-symmetry non-collinear spin ordering present in the bulk of an epitaxial antiferromagnetic thin film with an antiperovskite structure. Such unconventional torques can be robustly manipulated by controlling the antiferromagnetic ordering across the Néel temperature. This work provides essential insight into understanding how unconventional spin-orbit torques can arise in systems with lower crystalline or magnetic symmetry. This strategy of controlling the spin polarization direction by the design of the spin structure will lead to a much more efficient manipulation and deterministic switching of nano-magnets with arbitrary magnetization, as well as antiferromagnetic tunnel junctions^28^. In addition, our finding offers the possibility to design and control spin currents through manipulating the non-collinear spin order via strain, temperature, chemical doping, and possibly external excitation, opening new areas of research opportunities in antiferromagnetic spintronics^31–35^.

## Methods

### Sample growth, fabrication, and characterization

Epitaxial Mn_3_GaN thin films were grown on (001)-oriented LSAT substrates by DC reactive magnetron sputtering using a stoichiometric Mn_3_Ga target in a vacuum chamber with a base pressure of 1 × 10^−8^ Torr. During the growth, the Mn_3_GaN growth mode and surface crystalline structure were monitored by in situ reflection high energy electron diffraction (RHEED). The growth undergoes a 3D to 2D growth mode transition. The streaky RHEED pattern after the deposition implies a smooth film surface (Fig. 2a inset). The growth was performed at a substrate temperature of 550 °C and an Ar (62 sccm)/N_2_ (8 sccm) atmosphere of 10 mTorr. After the Mn_3_GaN growth, the sample was cooled down in vacuum. The Cu and Py thin films were subsequently sputter deposited at an Ar pressure of 3 mTorr. The atomically flat sample surface was verified using atomic force microscopy (Supplementary Fig. 1). We confirmed the thickness, epitaxial arrangement, and coherence of the Mn_3_GaN films using X-ray reflectivity, X-ray diffraction, and reciprocal space mappings. The growth rate of Py and Cu films were calibrated using X-ray reflectivity.

We patterned the samples by using photolithography followed by ion beam milling. Then 200 nm Pt/5 nm Ti electrodes were sputter deposited and defined by a lift-off procedure. Devices for ST-FMR were patterned into microstrips (20–50 μm wide and 40–100 μm long) with ground-signal-ground electrodes. Devices for electrical transport measurements were patterned into 100 μm wide and 500 μm long Hall bars.

### STEM measurements

The STEM sample was prepared through mechanical polishing down to a thickness of ~10 μm by using the precise polishing system (EM TXP, Leica). The polished specimen was then ion-milled using a 1–3 keV Ar ion beam (PIPS II, Gatan) to make the hole for the STEM observation. Afterwards, a low energy milling was performed using 0.1 keV Ar beam to minimize the surface damage from the prior ion-milling process.

The atomic structures were observed using a STEM (JEM-ARM200F, JEOL) at 200 kV equipped with an aberration corrector (ASCOR, CEOS GmbH). The optimum size of the electron probe was ~0.8 Å. The collection semi-angles of the HAADF detector were adjusted from 68 to 280 mrad in order to collect large-angle elastic scattering electrons for clear Z-sensitive images. The obtained raw images were processed with a band-pass Wiener filter with a local window to reduce a background noise (HREM research Inc.).

### ST-FMR measurements

During ST-FMR measurements, a microwave current at a fixed frequency was applied with the in-plane magnetic field swept from 0 to 0.2 T for driving the ferromagnetic layer Py through its resonance condition. The amplitude of the microwave current is modulated at a low frequency (1.713 kHz), and the mixing voltage is detected through a lock-in amplifier. For the low temperature ST-FMR measurements (including the room temperature results shown in Fig. 3), the device was wire bonded to a coplanar waveguide and then transferred into a liquid helium flow cryostat. For the high temperature measurements (Fig. 4), the sample is placed on a resistive heater with the device probed by the ground-signal-ground rf probe. The ST-FMR resonance line shape can be fitted to a sum of symmetric *V*_S_ and antisymmetric *V*_A_ Lorentzian components in the form $V_{{\mathrm{mix}}} = V_{{\mathrm{mix}},{\mathrm{S}}}\frac{{W^2}}{{(\mu _0H_{{\mathrm{ext}}} - \mu _0H_{{\mathrm{FMR}}})^2 + W^2}} + V_{{\mathrm{mix}},{\mathrm{A}}}\frac{{W(\mu _0H_{{\mathrm{ext}}} - \mu _0H_{{\mathrm{FMR}}})}}{{(\mu _0H_{{\mathrm{ext}}} - \mu _0H_{{\mathrm{FMR}}})^2 + W^2}}$, where *W* is the half-width-at-half-maximum resonance linewidth, *μ*_0_ is the permeability in vacuum and *H*_FMR_ is the resonance field. The in-plane *τ*_||_ and out-of-plane *τ*_⊥_ components are proportional to *V*_mix,S_ and *V*_mix,A_ components, which can be expressed as,

3 $$V_{{\mathrm{mix}},{\mathrm{S}}} = - \frac{{I_{{\mathrm{rf}}}}}{2}\left( {\frac{{dR}}{{d\varphi }}} \right)\frac{1}{{\alpha (2\mu _0H_{{\mathrm{FMR}}} + \mu _0M_{{\mathrm{eff}}})}}\tau _\parallel$$

4 $$V_{{\mathrm{mix}},{\mathrm{A}}} = - \frac{{I_{\mathrm{rf}}}}{2}\left( {\frac{{dR}}{{d\varphi }}} \right)\frac{{\sqrt {1 + M_{{\mathrm{eff}}}/H_{{\mathrm{FMR}}}} }}{{\alpha (2\mu _0H_{{\mathrm{FMR}}} + \mu _0M_{{\mathrm{eff}}})}}\tau _ \bot$$

where *I*_rf_ is the microwave current, *R* is the device resistance as a function of in-plane magnetic field angle *φ* due to the AMR of Py, *α* is the Gilbert damping coefficient, and *M*_eff_ is the effective magnetization. The AMR of Py is determined by measuring the device resistance as a function of magnetic field angle with a field magnitude of 0.1 T. We calibrate the microwave current *I*_rf_ by measuring the microwave current-induced device resistance change due to Joule heating^36,37^ (Supplementary Note 6). The in-plane and out-of-plane torques can be expressed as the angular dependence of the torque components with different spin polarization directions,

5 $$\tau _\parallel = \tau _{{\mathrm{x}},{\mathrm{AD}}}\sin \left( \varphi \right) + \tau _{{\mathrm{y}},{\mathrm{AD}}}\cos \left( \varphi \right) + \tau _{{\mathrm{z}},{\mathrm{FL}}}$$

6 $$\tau _ \bot = \tau _{{\mathrm{x}},{\mathrm{FL}}}\sin \left( \varphi \right) + \tau _{{\mathrm{y}},{\mathrm{FL}}}\cos \left( \varphi \right) + \tau _{{\mathrm{z}},{\mathrm{AD}}}$$

The strength of the torque components can then be determined from Eqs. (3–6) with the calibrated *I*_rf_ values, from which we noticed that the primary contribution to ${\mathbf{\tau }}_{{\mathrm{y}},{\mathrm{FL}}}$ is the current-induced Oersted field. The spin torque ratios can be expressed as,

7 $$\theta _x = \frac{{\tau _{{\mathrm{x}},{\mathrm{AD}}}}}{{\tau _{{\mathrm{y}},{\mathrm{FL}}}}}\frac{{{\mathrm{e}}\mu _0M_{\mathrm{s}}t_{{\mathrm{Py}}}t_{{\mathrm{MGN}}}}}{\hbar }$$

8 $$\theta _y = \frac{{\tau _{{\mathrm{y}},{\mathrm{AD}}}}}{{\tau _{{\mathrm{y}},{\mathrm{FL}}}}}\frac{{{\mathrm{e}}\mu _0M_{\mathrm{s}}t_{{\mathrm{Py}}}t_{{\mathrm{MGN}}}}}{\hbar }$$

9 $$\theta _z = \frac{{\tau _{{\mathrm{z}},{\mathrm{AD}}}}}{{\tau _{{\mathrm{y}},{\mathrm{FL}}}}}\frac{{{\mathrm{e}}\mu _0M_{\mathrm{s}}t_{{\mathrm{Py}}}t_{{\mathrm{MGN}}}}}{\hbar }$$

where *M*_s_ and *t*_Py_ are the saturation magnetization and the thickness of Py; *t*_MGN_ is the thickness of Mn_3_GaN. $\hbar$ is the reduced Planck’s constant and e is the electron charge. The saturation magnetization of Py was measured with SQUID magnetometry, and is indistinguishable from the effective magnetization determined by ST-FMR.

### Electrical transport measurements of Mn_3_GaN

Electrical transport measurements of Mn_3_GaN films were performed directly on as-grown films wire-bonded in a four-corner van der Pauw geometry. Both sheet resistance and Hall resistance were measured as a function of temperature and magnetic induction in a Quantum Design Physical Property Measurement System. Film resistivity was computed by solving the van der Pauw equation in conjunction with film thickness as measured with x-ray reflectivity, while Hall resistance was calculated by summing two orthogonal Hall configurations. The longitudinal resistivity and the Hall resistance of Mn_3_GaN films vs. temperature are reported in Supplementary Fig. 4.

### Temperature dependence of neutron diffraction

Single crystal neutron diffraction measurements were performed on the WISH time-of-flight diffractometer^38^ at ISIS, the UK neutron and muon source. A stack of eight, approximately 250 nm thick (001) Mn_3_GaN film samples with lateral dimensions 10 × 8 mm, were co-aligned and oriented for the measurement of nuclear and magnetic diffraction intensities in the (HK0) reciprocal lattice plane. The sample was first mounted within a ^4^He cryostat, and diffraction patterns were collected from a base temperature of 1.5 K up to 300 K, in 25 K steps. The sample was transferred to a medium-range furnace, and diffraction patterns were then collected at 320, 340, 360, and 390 K.

### Temperature dependence of X-ray diffraction

The X-ray diffraction data were acquired at beamline 6-ID-B at the Advanced Photon Source with 12 keV incident X-ray energy. The sample temperature was controlled employing an ARS high temperature cryostat. Data were collected with 5 K steps; and at each temperature the sample position was realigned with respect to the base-temperature reciprocal space matrix. The sample was mounted on a standard PSI Huber diffractometer. The representative temperature dependence of x-ray diffraction spectra around the LSAT (003) reflection can be found in Supplementary Fig. 2.

### Theoretical calculations

The electronic band structure of Mn_3_GaN was calculated by using first-principles density functional theory (DFT) with Quantum ESPRESSO^39^ and fully relativistic ultrasoft pseudopotentials^40^. The exchange and correlation effects were treated within the generalized gradient approximation (GGA)^41^. The plane-wave cut-off energy of 57 Ry and a 16 × 16 × 16 *k*-point mesh in the irreducible Brillouin zone were used in the calculations. Spin-orbit coupling and non-collinear Γ^5g^ antiferromagnetism were included in all electronic structure calculations. We note that even though the spin-orbit coupling in Mn_3_GaN is relatively small, it still plays an important role to couple the spin and the lattice, which lifts the spin rotation symmetry and allows the existence of the non-vanishing spin-Hall conductivity (Supplementary Note 10). The calculated band structures for Mn_3_GaN in antiferromagnetic and paramagnetic phases are shown in Supplementary Fig. 9.

The spin-Hall effect is given by^42^

10 $$\sigma _{ij}^k = \frac{{e^2}}{\hbar }{\int} {\frac{{d^3\vec k}}{{(2\pi )^3}}} \mathop {\sum}\nolimits_n {f_{n\vec k}{\mathrm{{\Omega} }}_{n,ij}^k\left( {\vec k} \right)}$$

11 $${\Omega} _{n,ij}^k(\vec k) = - 2{\it{Im}}\mathop {\sum}\nolimits_{n \neq n^{\prime}} {\frac{{\left\langle {n\vec k\left| {J_i^k} \right|n^{\prime}\vec k} \right\rangle \left\langle {n^{\prime}\vec k\left| {v_j} \right|n^{\prime}\vec k} \right\rangle }}{{\left( {E_{n\vec k} - E_{n^{\prime}\vec k}} \right)^2}}}$$

where $f_{n\vec k}$ is the Fermi-Dirac distribution for the *n*th band, $J_i^k = \frac{1}{2}\{ v_i,s_k\}$ is the spin current operator with spin operator *S*_*k*_, $v_j = \frac{1}{\hbar }\,\frac{{\partial H}}{{\partial k_j}}$ is the velocity operator, and $i,j,k = x,y,z$. ${\mathrm{{\Omega} }}_{n,ij}^k\left( {\vec k} \right)$ is referred to as the spin Berry curvature in analogy to the ordinary Berry curvature. In order to calculate the spin- Hall conductivities, we construct the tight-binding Hamiltonians using PAOFLOW code^43^ based on the projection of the pseudo-atomic orbitals (PAO)^44,45^ from the non-self-consistent calculations with a 16 × 16 × 16 *k*-point mesh. The spin-Hall conductivities were calculated using the tight-binding Hamiltonians with a 48 × 48 × 48 *k*-point mesh by the adaptive broadening method to get the converged values.

### Synchrotron spectroscopy and microscopy

X-ray magnetic circular dichroism (XMCD) and X-ray magnetic linear dichroism (XMLD) spectroscopy were measured at beamline 4.0.2, and X-ray microscopy at beamline 11.0.1.1 (PEEM-3) at the Advanced Light Source (ALS). In spectroscopy, total-electron-yield mode was employed by monitoring the sample drain current, and a grazing incidence angle of 30° to the sample surface along the [110] direction to probe the magnetic state. The obtained dichroism energies giving information on the magnetic nature of the Mn_3_GaN were then used to image the domain texture by X-ray photoemission electron microscopy (XPEEM), also taken with X-rays at a 30° grazing incidence along the [110] direction. Images taken at the maximum dichroism energies as a function of polarization were normalized by pre-edge energy images in order to minimize any topographic and work function contrast while emphasizing the magnetic contrast of the Mn_3_GaN films.

## Supplementary information

Supplementary Information

## Data Availability

The data that support the findings of this study are available from the corresponding author on reasonable request.
