# Supplementary material for: Controlling spin current polarization through non-collinear antiferromagnetism
Source: Nat Commun. 2020 Sep 16;11:4671. doi: 10.1038/s41467-020-17999-4 (PMC7494910; doi:10.1038/s41467-020-17999-4)
Supplement: Supplementary file 1 — Supplementary Information [file 41467_2020_17999_MOESM1_ESM.pdf]

Supplementary Information for

Controlling spin current polarization through non-collinear antiferromagnetism

T. Nan[1], C. X. Quintela[1], J. Irwin[2], G. Gurung[3], D. F. Shao[3], J. Gibbons[4], N. Campbell[2], K. Song[5], S. Y. Choi[6], L. Guo[1], R. D. Johnson[7,8,9], P. Manuel[9], R. V. Chopdekar[10], I. Hallsteinsen[10,11], T. Tybell[11], P. J. Ryan[12,13], J. W. Kim[12], Y. S. Choi[12], P. G. Radaelli[7], D. C. Ralph[4,14], E. Y. Tsymbal[3], M. S. Rzchowski[2], C. B. Eom[1*]

[1]Department of Materials Science and Engineering, University of Wisconsin-Madison, Madison, Wisconsin 53706, USA; [2]Department of Physics, University of Wisconsin-Madison, Madison, Wisconsin 53706, USA; [3]Department of Physics and Astronomy & Nebraska Center for Materials and Nanoscience, University of Nebraska, Lincoln, NE 68588, USA; [4]Laboratory of Atomic and Solid State Physics, Cornell University, Ithaca, New York 14853, USA; [5]Department of Materials Modeling and Characterization, KIMS, Changwon 51508, South Korea; [6]Department of Materials Science and Engineering, POSTECH, Pohang 37673, South Korea; [7]Clarendon Laboratory, Department of Physics, University of Oxford, Parks Road, Oxford OX1 3PU, UK; [8]ISIS Facility, Rutherford Appleton Laboratory, Chilton, Didcot, OX11 0QX, UK; [9]Department of Physics and Astronomy, University College London, Gower Street, London, WC1E 6BT, UK; [10]Advanced Light Source, Lawrence Berkeley National Laboratory (LBNL), 1 Cyclotron Road, Berkeley, California 94720, USA; [11]Department of Electronic Systems, Norwegian University of Science and Technology, Trondheim 7491, Norway; [12]Advanced Photon Source, Argonne National Laboratory, Argonne, Illinois 60439, USA; [13]School of Physical Sciences, Dublin City University, Dublin 11, Ireland; [14]Kavli Institute at Cornell for Nanoscale Science, Ithaca, New York 14853, USA

* Corresponding author. Email: eom@engr.wisc.edu

Supplementary Note 1: Structure and surface characterization of Py/Mn_3GaN/LSAT(001) heterostructures

In Fig. 2a of the main text, we show the lab source out-of-plane x-ray diffraction of the Mn_3GaN (002) peak. In the 2θ-ω scan with wide 2θ angles, we did not find any additional diffraction peak other than the (00*l*) reflections. Supplementary Figure 1a shows the rocking curve of the Mn_3GaN (002) peak, where the full-width-half-maximum value is ~0.02° indicating the high crystalline quality of the film. The azimuthal Φ-scan around the (022) reflection (Supplementary Figure 1b) indicates that the Mn_3GaN films grew epitaxially with a cube-on-cube alignment with the underlying LSAT substrate. The in-plane (a=3.916 Å) and out-of-plane (c=3.891 Å) lattice parameters of Mn_3GaN were determined from the x-ray reciprocal space mapping (RSM) measurements around the (-113) LSAT peak (bulk cubic Mn_3GaN lattice parameter of 3.898 Å), which gives rise to a tetragonal lattice. The tetragonal Mn_3GaN thin film breaks the cubic symmetry, potentially inducing a net magnetic moment[1], which is evidenced by the presence of an anomalous Hall effect near 100 K (Supplementary Figure 4b). Supplementary Figure 1c shows atomic force microscopy image of the final surface of the 10 nm Py/2nm Cu/20 nm Mn_3GaN/LSAT sample indicating an atomically flat surface with a RMS roughness <3 Å.

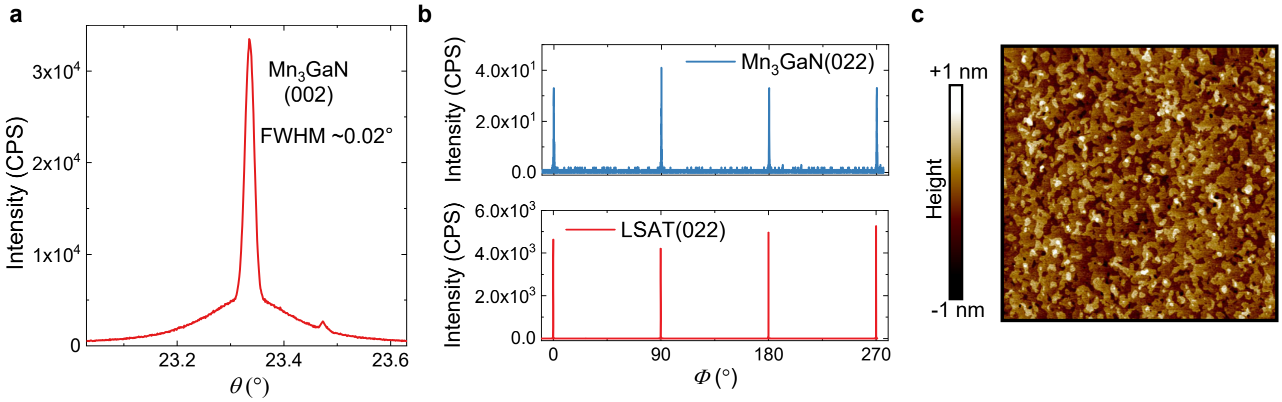

Supplementary Figure 1. a, Rocking curve of the (002) Mn_3GaN peak. **b**, Φ-scan around the Mn_3GaN peak showing the epitaxial arrangement with the underlying LSAT substrate. **c**, Atomic force microscopy images of the heterostructure: 10 nm Py/20 nm Mn_3GaN on LSAT (001) substrate.

Supplementary Note 2: Temperature dependence of x-ray diffraction

Bulk Mn_3GaN shows large negative thermal expansion behavior, which is linked to the first-order magnetic phase (Néel) transition. To identify the magneto-structural transition and thus determine the Néel temperature T_N of our Mn_3GaN thin films, we performed x-ray diffraction experiments as a function of temperature. The temperature dependence of the out-of-plane lattice parameter was derived from the evolution of the Mn_3GaN (003) reflection, as shown in Supplementary Figure 2.

The observed elevated T_N in our Mn_3GaN thin films is due to slight nitrogen deficiency and the epitaxial strain induced tetragonal distortion. It has been documented experimentally in bulk polycrystalline samples that Nitrogen deficiency increases T_N (Ref. 2). It has also been reported from computational study that for effective-tensile strain (equivalent to a tetragonality c/a<1) T_N increases[3], in which we got c/a=~0.996 in our films (determined from RSM and STEM). Finally, we note that grain boundaries in polycrystalline samples can suppress the large-scale fluctuations associated with phase transitions, lowering the transition temperature.

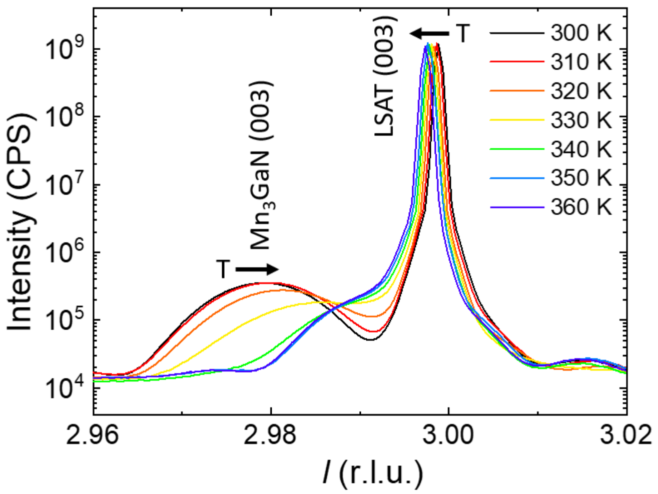

Supplementary Figure 2. Temperature dependence of x-ray *l* scans around the LSAT (003) reflection.

Supplementary Note 3: Neutron diffraction of Mn_3GaN thin films

Single crystal neutron diffraction measurements were performed on a stack of eight, approximately 250 nm thick (001) Mn_3GaN film samples with lateral dimensions 10 x 8 mm, co-aligned and oriented for the measurement of nuclear and magnetic diffraction intensities in the (HK0) reciprocal lattice plane (see Methods section of the main text). Weak diffraction peaks were observed in the proximity of the strong (100) and (110) substrate Bragg peaks, as shown in Supplementary Figure 3 left. These weak peaks were found at positions corresponding to longer d-spacing values than the substrate reflections, consistent with the lattice mismatch between the Ma_3GaN film and substrate observed using x-ray diffraction. The temperature dependence of the integrated intensities of the weak peak is plotted in Supplementary Figure 3 right. Critical behavior was found for both intensities at T_N ~340 K, which is consistent with the magnetic phase transition of the Ma_3GaN films observed in other measurements. Taken together, these observations allowed us to robustly assign the weak diffraction intensities to the Ma_3GaN film, with a magnetic component below T_N. Furthermore, calculations of the nuclear scattering intensity from Ma_3GaN confirmed that the (100) nuclear intensity is ~0, whilst the (110) is bright, as seen at 390 K.

The fact that the magnetic diffraction intensity coincided exactly with the nuclear intensity confirmed that the magnetic structure of the Ma_3GaN film below T_N had a Γ-point propagation vector. Rigorous searches for diffraction peaks corresponding to other propagation vectors within the first Brillouin zone, as well as the monotonic behavior of the diffraction intensities, indicated that the Γ-point magnetic structure was the only one present at all measured temperatures below $T_{N,}$ within the detection limit of the experiment.

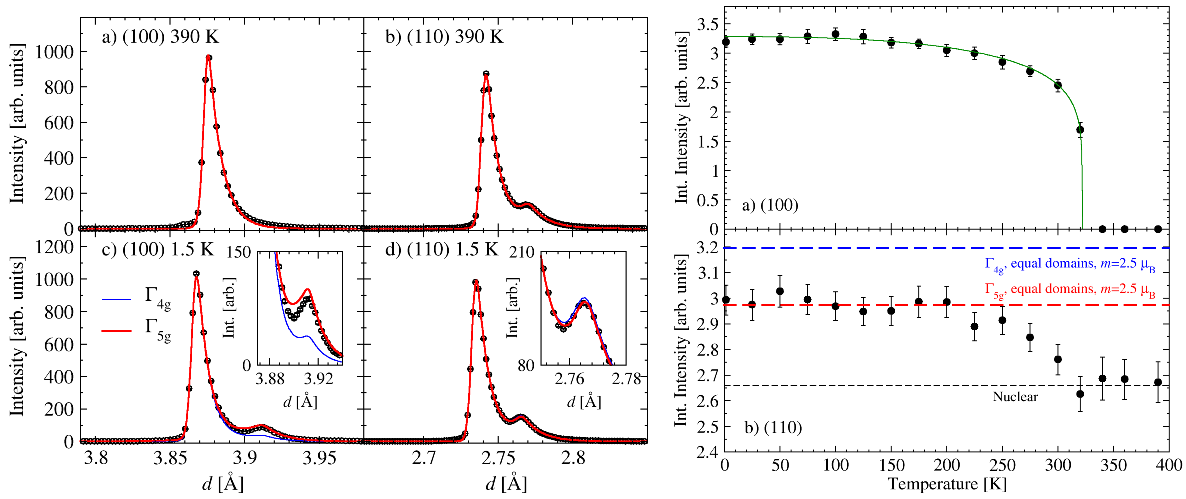

Supplementary Figure 3. **Left**: Neutron diffraction data from a stack of 8 250-nm Mn_3GaN films on LSAT (001) substrates. The strong reflections on the left side are from the (100) **a**, **c** and (110) **b**, **d** Bragg peaks of the substrates. The side peak seen at 390 K in **b** is the (110) nuclear peak from the films. A side peak is absent in **a** because the (100) nuclear intensity from the film is ~0. Magnetic Bragg peaks at the Γ point (i.e., on top of the nuclear peaks of the film) develop at low temperatures **c**, **d**. Solid circles are experimental data, fitted with the Γ_{4g} (blue lines) and Γ_{5g} (red lines) representations. **Right**: The temperature dependence of the integrated intensity as a function of temperature for the (100) **a** and (100) **b** magnetic peaks. The dashed lines in **b** are the predictions of the magnetic intensity for the Γ_{4g} (blue) and Γ_{5g} (red) representations (see text). The green line in **a** is a guide to the eye. The error bars indicate fitting uncertainties.

Symmetry analyses using both the little group of the propagation vector, and the full Γ-point magnetic representation, were performed for the relevant Wyckoff positions using BasIreps (part of the FullProf package[4]) and Isodistort[56], respectively. Two three-dimensional irreducible representations appear in the decomposition of the full magnetic representation, typically labelled Γ^{4g} and Γ^{5g}. Symmetry-distinct directions of the magnetic order parameter in the space spanned by both irreducible representations leads to 12 different magnetic symmetries. However, under the assumption that the magnetic structure has zero net magnetic moment (as evidenced by bulk magnetometry), and that every manganese site has the same moment magnitude, these 12 symmetries are reduced to just two with magnetic space groups *R*-3*m*' and *R*-3*m,* which transform according to Γ^{4g} and Γ^{5g}, respectively. In both symmetries, magnetic moments are aligned within the (111) crystallographic plane forming 120° triangular motifs. In the former case, moments lie within the mirror planes, and in the latter, perpendicular to the mirror planes. In fact, both magnetic structures are related by a 90° global rotation of spins in spin space, making them difficult to differentiate in diffraction, as discussed below.

The magnetic diffraction patterns for both Γ^{4g} and Γ^{5g} magnetic structures were calculated using FullProf[4] and used to fit the diffraction data (blue and red lines in Supplementary Figure 3 left, respectively). In both cases we assumed a Mn magnetic moment of 2.5 μ_B and equal population of all possible antiferromagnetic domains – a good assumption as the neutron diffraction experiment was performed using a stack of eight films, and the neutron beam illuminated the full volume of every film. It is clear that the Γ_{5g} magnetic structure model is most consistent with the measured magnetic diffraction data, especially at the (100) reciprocal lattice point (Supplementary Figure 3 left). The sensitivity of the (110) reflection is most apparent when considering the relative magnitude of the magnetic intensity compared to the nuclear, as shown by the black, blue and red dashed lines in Supplementary Figure 3 right.

In summary, single crystal neutron diffraction experiments on our 250 nm thick Mn_3GaN thin film stack demonstrated that long-range Γ-point ordering of manganese magnetic moments occurred below T_N =340 K, and that the magnetic diffraction intensities at all measured temperatures below T_N were fully consistent with the Γ_{5g} magnetic structure previously proposed for bulk Mn_3GaN.

Supplementary Note 4: Electrical transport properties of Mn_3GaN thin films

We measure as-grown 5 mm x 5 mm Mn_3GaN thin films on LSAT in a Quantum Design PPMS in a van der Pauw geometry. Resistivity versus temperature data shown in Supplementary Figure 4a indicate that Mn_3GaN is metallic. The slope of the Hall resistivity vs. field shows a sign change around 200 K. In semiconductors, such a sign change should be accompanied with a nonlinear Hall signal. However, as Mn_3GaN is known to be highly covalent with many bands crossing the Fermi energy, none with particularly high mobility, the Hall curves are linear in field, and the sign change is caused by subtle band population changes with temperature. We also observed a small hysteresis in the Hall measurement around 100 K (red curve in Supplementary Figure 4b), indicating a presence of an anomalous Hall effect. The connection between ordinary Hall coefficient and magnetism allows us to confirm the Néel temperature at around 350 K by a flattening out of R_H versus temperature (Supplementary Figure 4c).

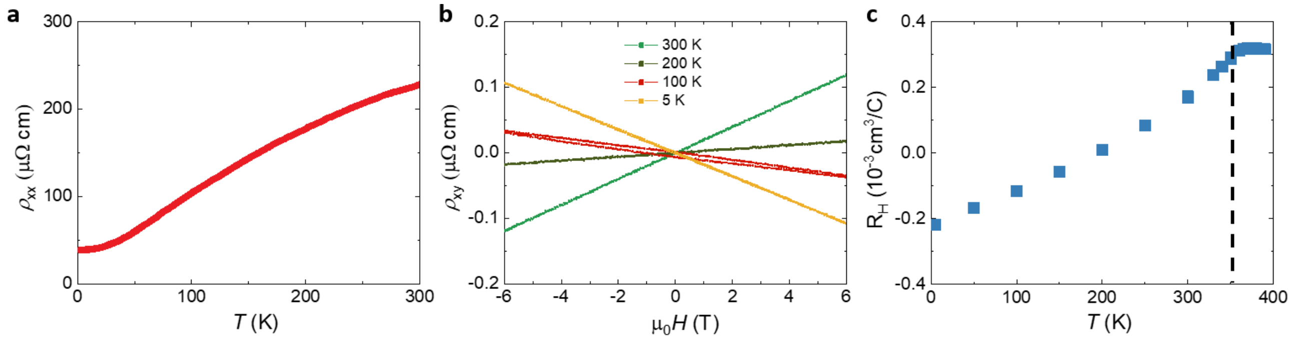

Supplementary Figure 4. **a**, Longitudinal resistivity ρ_{xx} vs. temperature curve for a 20 nm Mn_3GaN thin film on LSAT (001) substrate, showing metallic behaviour. **b**, Hall resistivity ρ_{xy} vs. out-of-plane magnetic field at various representative temperatures. **c**, Temperature dependence of ordinary Hall coefficient R_H, indicating a transition temperature at 350 K (dashed line).

Supplementary Note 5: Magnetic properties of Mn_3GaN thin films

We measure as-grown 5mm x 5mm Mn_3GaN thin films on LSAT in a Quantum Design MPMS 3 with an in-plane applied field. The substrate contribution to the magnetization was measured separately and subtracted based on magnetic impurity density. Magnetization versus temperature data shown, in Supplementary Figure 5, have two clear transitions. The first is around 350 K, where the zero-field-cooled and field-cooled curves deviate. We ascribe this to the Néel Transition and note that the temperature matches with the flattening of the R_H temperature dependence in Fig S4c. The second transition is around 200 K, and corresponds with a distinct further deviation of the field-cooled curve from the zero-field-cooled curve. This transition corresponds to the onset of anomalous Hall effect, shown partly in Fig S4b. Bulk work on Mn_3GaN does not show any evidence for a new phase or net-moment character onset around 200 K, suggesting that this signal may not be due to an intrinsic or bulk mechanism.

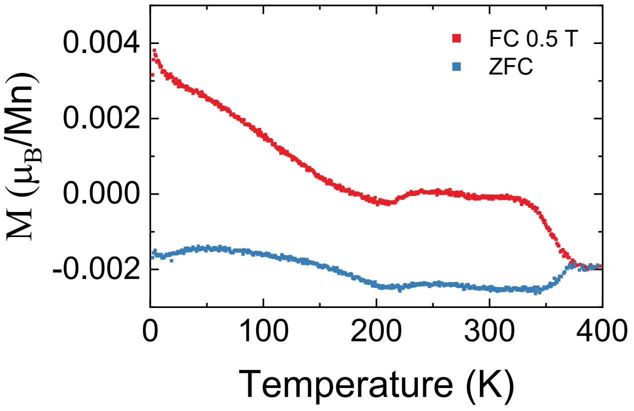

Supplementary Figure 5. Net magnetization vs temperature curves for field-cooled (blue) and zero-field-cooled (red) samples. Distinct transitions are visible at 350K and 200K, and ascribed to the Neel temperature and the onset of the anomalous Hall effect respectively. The slight vertical offset comes from different ratios of magnetic impurities to intrinsic diamagnetism between the film substrate and reference substrate pieces.

Supplementary Note 6: ST-FMR line shape analysis

The ST-FMR signal with the current-induced in-plane and out-of-plane torque components is modeled by the Landau–Lifshitz–Gilbert–Slonczewski equation[7]. The ST-FMR mixing voltage can be then written in the form as,

$$V_{\text{mix}} = V_{\text{mix,S}}\frac{W^2}{(\mu_0 H_{\text{ext}}-\mu_0 H_{\text{FMR}})^2+W^2} + V_{\text{mix,A}}\frac{W(\mu_0 H_{\text{ext}}-\mu_0 H_{\text{FMR}})}{(\mu_0 H_{\text{ext}}-\mu_0 H_{\text{FMR}})^2+W^2} \quad \text{(S1)}$$

where W is the half-width-at-half-maximum resonance linewidth, μ_0 is the permeability in vacuum and H_{FMR} is the resonance field. The symmetric $V_{\text{mix,S}}$ and the antisymmetric $V_{\text{mix,A}}$ Lorentzian amplitudes, which are proportional to the in-plane $\tau_{\parallel}$ and out-of-plane torque $\tau_{\perp}$ components, can be written as

$$V_{\text{mix,S}} = -\frac{I_{\text{rf}}}{2}\left(\frac{dR}{d\varphi}\right)\frac{1}{\alpha(2\mu_0 H_{\text{FMR}}+\mu_0 M_{\text{eff}})}\tau_{\parallel} \quad \text{(S2)}$$

$$V_{\text{mix,A}} = -\frac{I_{\text{rf}}}{2}\left(\frac{dR}{d\varphi}\right)\frac{\sqrt{1+M_{\text{eff}}/H_{\text{FMR}}}}{\alpha(2\mu_0 H_{\text{FMR}}+\mu_0 M_{\text{eff}})}\tau_{\perp} \quad \text{(S3)}$$

where I_{rf} is the microwave current, R is the device resistance as a function of in-plane magnetic field angle φ due to the AMR of Py, α is the Gilbert damping coefficient, and M_{eff} is the effective magnetization. The microwave current I_{rf} with given microwave power is calibrated by measuring the device resistance change due to Joule heating effect[8,9]. We can compare the change of device resistance induced by the applied microwave power to that induced by the injection of a dc current I_{dc}. The rf current I_{rf} can then be determined as $I_{\text{rf}} = \sqrt{2}I_{\text{dc}}$, since Joule heating from ac and dc current are $\frac{1}{2}{I_{\text{rf}}}^2R$ and ${I_{\text{dc}}}^2R$. Supplementary Figure 6a shows the resistance change for a typical device (10 nm Py/2nm Cu/20 nm Mn_3GaN/LSAT) as a function of dc current and rf power (at 7 GHz). To calibrate the anisotropic magnetoresistance $R(\varphi)$, we measure the device resistance as a function of magnetic field angle by rotating an in-plane magnetic field of 0.1 T produced by a rotary electromagnet. Supplementary Figure 6b shows the $dR/d\varphi$ as a function of magnetic field angle φ. The magnetic resonance properties were characterized by the frequency dependence of ST-

FMR measurements. Supplementary Figure 6c shows the resonance linewidth W as a function of frequency f. The Gilbert damping coefficient is calculated from $\alpha = \frac{|\gamma|}{2\pi f}(W - W_0)$, where W_0 is the inhomogeneous linewidth broadening, and γ is the gyromagnetic ratio. From the linear fitting in Supplementary Figure 6c, we obtained $\alpha = 0.008$. The effective magnetization M_{eff} is obtained by a fit of the resonance field $\mu_0 H_{FMR}$ as a function of frequency to the Kittel equation, $\mu_0 H_{\text{FMR}} = \frac{1}{2}\left[-\mu_0 M_{\text{eff}} + \sqrt{(\mu_0 M_{\text{eff}})^2 + 4\left(\frac{f}{\gamma}\right)^2}\right] - \mu_0 H_{\text{k}}$, where $\mu_0 H_k$ is the in-plane anisotropy field. As shown in Supplementary Figure 6d, the effective magnetization M_{eff} is found to be 7.2×10^5 A/m.

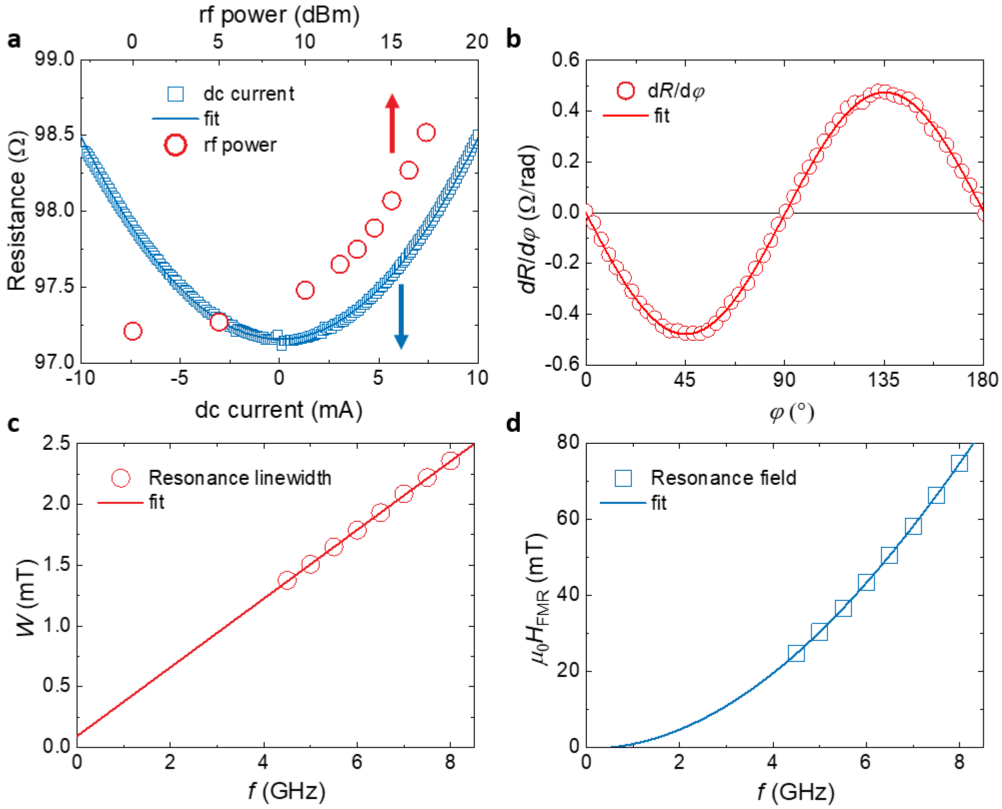

Supplementary Figure 6. a, Resistance change as a function of applied dc current (blue) and microwave current (red), induced in a device (10 nm Py/2nm Cu/20 nm Mn_3GaN/LSAT) due to Joule heating. **b,** $dR/d\varphi$ vs. the magnetic field angle φ derived from the anisotropic magnetoresistance of the same sample. **c,** The resonance linewidth W as a function of frequency f. The solid curve shows the fit to a linear function, which gives a Gilbert damping coefficient of $\alpha = 0.008$. **d**, Dependence of the resonance field $\mu_0 H_{\text{FMR}}$ upon frequency f. The data is fitted to the Kittel equation. The error bars, smaller than the size of the symbols, indicate fitting uncertainties.

Supplementary Note 7: Angular dependent ST-FMR at different temperatures

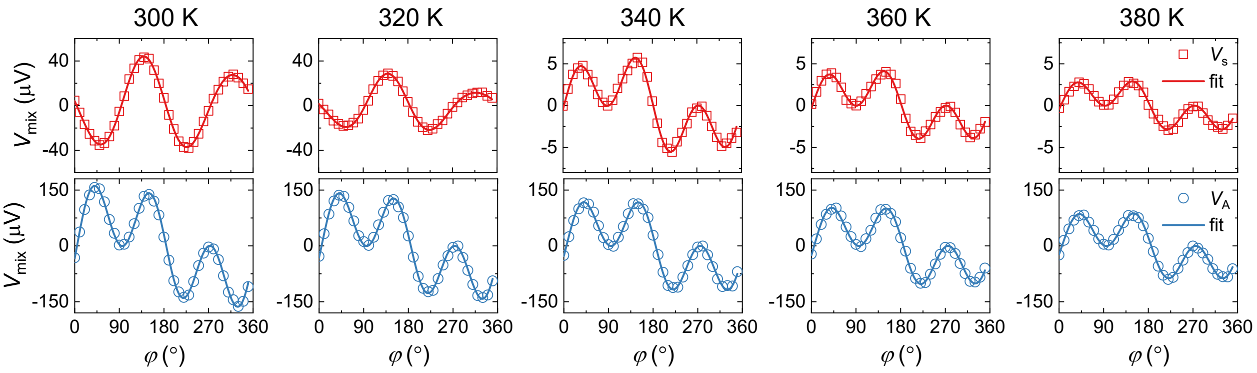

Supplementary Figure 7. Angular dependence of the ST-FMR symmetric $V_{\mathrm{mix,s}}$ (red) and the antisymmetric $V_{\mathrm{mix,A}}$ (blue) components at different temperatures from 300 to 380 K, from which we extracted the temperature dependence of the torque ratios (shown in Fig. 4 of the main text). The error bars, smaller than the size of the symbols, indicate fitting uncertainties.

Supplementary Note 8: ST-FMR measurements at low temperatures

Supplementary Figure 8 shows the temperature dependence of the spin torque ratios θ_y, θ_x and θ_z in the low temperature range 30-300 K. Interestingly, the conventional spin torque ratio θ_y (Supplementary Figure 8a) changes sign at ~200-250 K, which may correspond to the sign change of the Hall coefficient in Mn_3GaN at ~ 200 K (Supplementary Figure 4c). The amplitude of the unconventional torques θ_x (Supplementary Figure 8b) and θ_z (Supplementary Figure 8c) both decrease with decreasing temperature, which could be attributed to the increase of the canted moment in Mn_3GaN (Supplementary Figure 5).

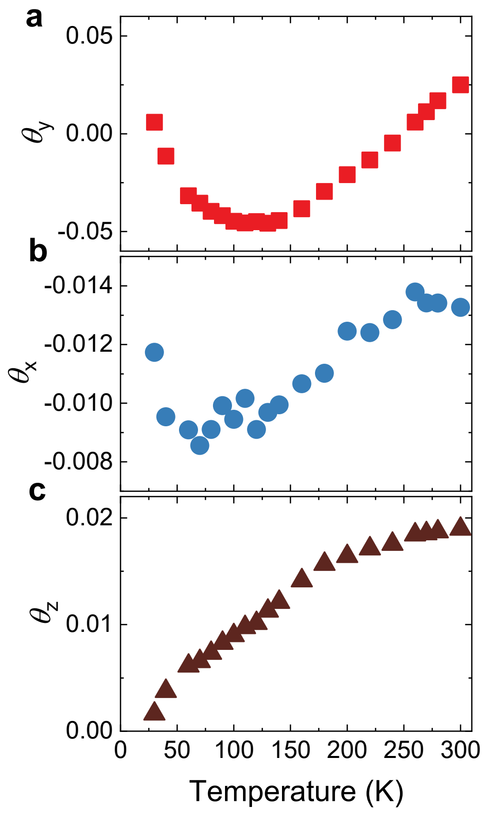

Supplementary Figure 8. a-c, Spin torque ratios θ_y, θ_x and θ_z as a function of temperatures.

Supplementary Note 9: Theory calculations

Supplementary Figure 9 shows the electronic band structure of Mn_3GaN for different magnetic phases. In the paramagnetic phase, Mn_3GaN has a space group of $Pm\bar{3}m$ (#221). When the Γ^{5g} non-collinear antiferromagnetism is present below T_N, the symmetry of Mn_3GaN is reduced to $R\bar{3}m$. The band structure of Γ^{5g} phase is shown in Supplementary Figure 9b. The changes of symmetry due to magnetism significantly influence the electronic structure of Mn_3GaN, which also leads to the difference in spin-Hall conductivity between the Γ^{5g} and the paramagnetic phases.

Table S1 summarizes the theoretical and calculated spin-Hall conductivity tensors derived from different Mn_3GaN magnetic phases. As mentioned above and in the main text, paramagnetic Mn_3GaN with the high-symmetry space group $Pm\bar{3}m$ only allows the conventional components in spin-Hall conductivity tensors[10]. The reduction of symmetry due to the non-collinear spin structure allows the unconventional components in spin-Hall conductivity tensors[10], which is consistent with our observation of unconventional spin-orbit torque.

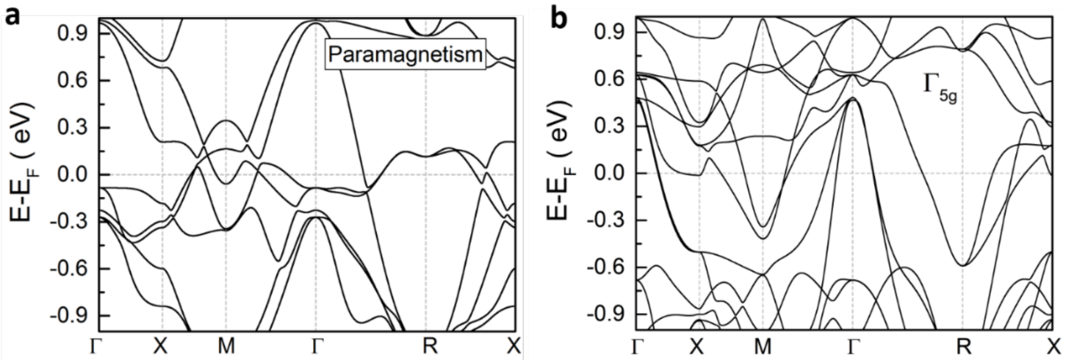

Supplementary Figure 9. **a, b,** The electronic band structures of Mn_3GaN in the paramagnetic (**a**) and in the antiferromagnetic Γ^{5g} phases (**b**).

Supplementary Table 1: The theoretical and calculated spin-Hall conductivity tensor for Γ^{5g} phase and paramagnetic phase in Mn_3GaN.

		σ^x	σ^y	σ^z
Γ^{5g}	Tensor shape	$\begin{bmatrix} 0 & -\sigma^{y}_{yx} & \sigma^{y}_{yx} \\ \sigma^{x}_{yx} & -\sigma^{y}_{xx} & -\sigma^{z}_{yx} \\ -\sigma^{x}_{yx} & \sigma^{z}_{yx} & \sigma^{y}_{xx} \end{bmatrix}$	$\begin{bmatrix} \sigma^{y}_{xx} & -\sigma^{x}_{yx} & \sigma^{z}_{yx} \\ \sigma^{y}_{yx} & 0 & -\sigma^{y}_{yx} \\ -\sigma^{z}_{yx} & \sigma^{x}_{yx} & -\sigma^{y}_{xx} \end{bmatrix}$	$\begin{bmatrix} -\sigma^{y}_{xx} & -\sigma^{z}_{yx} & \sigma^{x}_{yx} \\ \sigma^{z}_{yx} & \sigma^{y}_{xx} & -\sigma^{x}_{yx} \\ -\sigma^{y}_{yx} & \sigma^{y}_{yx} & 0 \end{bmatrix}$
	Calculated tensor	$\begin{bmatrix} 0 & 45 & -45 \\ -29 & 40 & 114 \\ 29 & -114 & -40 \end{bmatrix}$	$\begin{bmatrix} -40 & 29 & -114 \\ -45 & 0 & 45 \\ 114 & -29 & 40 \end{bmatrix}$	$\begin{bmatrix} 40 & -114 & -29 \\ -114 & -40 & 29 \\ 45 & -45 & 0 \end{bmatrix}$
Paramagnetic	Tensor shape	$\begin{bmatrix} 0 & 0 & 0 \\ 0 & 0 & -\sigma^{z}_{yx} \\ 0 & \sigma^{z}_{yx} & 0 \end{bmatrix}$	$\begin{bmatrix} 0 & 0 & \sigma^{z}_{yx} \\ 0 & 0 & 0 \\ -\sigma^{z}_{yx} & 0 & 0 \end{bmatrix}$	$\begin{bmatrix} 0 & -\sigma^{z}_{yx} & 0 \\ \sigma^{z}_{yx} & 0 & 0 \\ 0 & 0 & 0 \end{bmatrix}$
	Calculated tensor	$\begin{bmatrix} 0 & 0 & 0 \\ 0 & 0 & 41 \\ 0 & -41 & 0 \end{bmatrix}$	$\begin{bmatrix} 0 & 0 & -41 \\ 0 & 0 & 0 \\ 41 & 0 & 0 \end{bmatrix}$	$\begin{bmatrix} 0 & 41 & 0 \\ -41 & 0 & 0 \\ 0 & 0 & 0 \end{bmatrix}$

Supplementary Note 10: The role of spin-orbit coupling on spin Hall effect

The spin-Hall conductivities above are obtained from the calculation with the spin-orbit coupling. However, the spin-Hall effect is suggested to exist in non-collinear magnets even with the absence of spin-orbit coupling[11,12]. Since Mn_3GaN is a non-collinear antiferromagnet composed by the light elements Mn, Ga, and N, figuring out the role of the relatively small spin-orbit coupling on the spin-Hall effect will help us to have a better understanding of the unconventional spin-orbit torque we observed.

Here we analyze the symmetry of Mn_3GaN when the spin-orbit coupling is absent. In this case the spin is not directly coupled to the lattice. Therefore, spin rotation symmetry operations become the symmetry operations of the system. As a result, they transform the spin Berry curvature in the same way as they do the spin itself. For example, we consider the 180° spin rotation around *z* direction, S_{2z}, which reverses the sign of the spin components along the *x* and *y* directions, but does not change the spin component along the *z* direction. The transformation of the spin Berry curvature under S_{2z} is as follows:

$$S_{2z}\Omega^x_{\alpha\beta}(\vec{k}) = -\Omega^x_{\alpha\beta}(\vec{k}),$$

$$S_{2z}\Omega^y_{\alpha\beta}(\vec{k}) = -\Omega^y_{\alpha\beta}(\vec{k}),$$

$$S_{2z}\Omega^z_{\alpha\beta}(\vec{k}) = \Omega^z_{\alpha\beta}(\vec{k}).$$

On the other hand, the spin Berry curvature is even under the time reversal symmetry operation:

$$T\Omega^\gamma_{\alpha\beta}(\vec{k}) = \Omega^\gamma_{\alpha\beta}(-\vec{k}).$$

Figs. S10a and b show transformation of the Mn magnetic moments in the (111) Ga-Mn plane of Mn_3GaN in the AFM Γ_{5g} phase under the S_{2z} and *T* symmetry operations (here the *z* axis is set along the [111] direction). It is seen that the magnetic structures are not identical before and after the transformations, indicating that the S_{2z} and *T* symmetries are broken in Mn_3GaN. However, the system is invariant under the product of S_{2z} and *T*, as shown in Supplementary Figure 10c.

The transformation of the spin Berry curvature under the symmetry preserved TS_{2z} is as follows:

$$TS_{2z}\Omega^x_{\alpha\beta}(\vec{k}) = -\Omega^x_{\alpha\beta}(-\vec{k}),$$

$$TS_{2z}\Omega^y_{\alpha\beta}(\vec{k}) = -\Omega^y_{\alpha\beta}(-\vec{k}),$$

$$TS_{2z}\Omega^z_{\alpha\beta}(\vec{k}) = \Omega^z_{\alpha\beta}(-\vec{k}).$$

This means for the $\Omega^x_{\alpha\beta}$ and $\Omega^y_{\alpha\beta}$ are odd under TS_{2z}. Since the spin Hall conductivity is determined by the integration of the spin Berry curvature over the entire Brillouin zone, this property leads to zero values for the spin Hall conductivity tensor elements $\sigma^x_{\alpha\beta}$ and $\sigma^y_{\alpha\beta}$. On the other hand, the $\Omega^z_{\alpha\beta}$ is even under TS_{2z}. Therefore, the spin Hall conductivity tensor element, $\sigma^z_{\alpha\beta}$, with the spin polarization along the *z* direction is not affected by this symmetry. However, in Mn_3GaN, there are three mirror symmetry reflections parallel to the *z* direction (Supplementary Figure 10d), which reverse the spin component along the *z* direction and reverse the sign of $\Omega^z_{\alpha\beta}$, for example:

$$M_x\Omega^z_{\alpha\beta}(k_x, k_y, k_z) = -\Omega^z_{\alpha\beta}(-k_x, -k_y, k_z).$$

These mirror symmetries enforce $\sigma^z_{\alpha\beta}$ to be zero.

Our conclusion about the spin Hall conductivity tensor to be zero for Mn_3GaN in the AFM Γ_{5g} phase in the absence of spin-orbit coupling is consistent with that obtained using the linear-response-symmetry code[13]. Thus, we conclude that even though the spin-orbit coupling in Mn_3GaN is relatively small, it still plays an important role to couple the spin and the lattice, which lifts the spin rotation symmetry and allows the existence of the non-vanishing spin Hall conductivity.

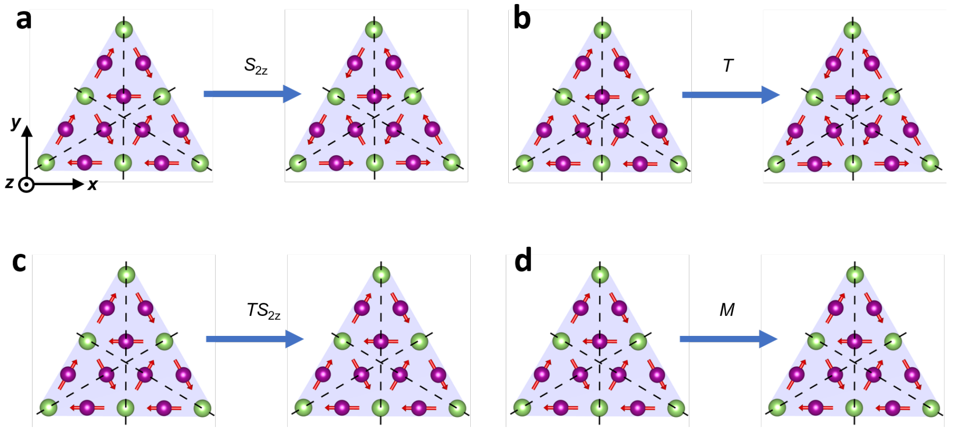

Supplementary Figure 10. Transformation of the Mn magnetic moments in the (111) Ga-Mn plane of Mn_3GaN in the AFM Γ_{5g} phase under symmetry operations. Here the *z* axis is set along the [111] direction. **a**, The 180° spin rotation around *z* direction, S_{2z}. **b**, The time reversal symmetry operation, *T*. **c**, The combined symmetry operation, TS_{2z}. **d**, The mirror symmetry reflections, *M*, perpendicular to the [111] plane (denoted by the black dashed lines).

Supplementary Note 11: Influence of antiferromagnetic domains

In magnets, symmetry generally requires the existence of degenerate domains, which strongly influence the properties of materials. For example, in noncollinear antiferromagnet Mn_3Ge and Ir_3Mn, applying time reversal symmetry operation can reverse the moments and generate degenerate domains with opposite chirality, thus eliminating the anomalous Hall effect that is odd under time reversal symmetry[14]. Similar domains are also possible in our Mn_3GaN film. The time reversal symmetry operation does not influence the spin-Hall conductivity. However, degenerate domains can be obtained by other symmetry operations. For example, a four-fold rotation symmetry around the z direction leads to four degenerate domains, denoted as D1 to D4 in Supplementary Figure 11. The elements of the spin Hall conductivity tensors of D_k (k=1,2,3,4) can be transformed from each other according to

$$\sigma^{s}_{ij,[D_k]} = \sum_{l,m,n} R_{z_{il}} R_{z_{jm}} R_{z_{kn}}\, \sigma^{s}_{lm,[D_n]},$$

Where $R_{z_{il}}$ is an element of the rotation matrix R_z. Therefore, the average spin Hall conductivities can be obtained as

$$\bar{\sigma}^{s}_{ij} = \frac{1}{4} \sum_k \sigma^{s}_{ij,[D_k]}.$$

We found that if the four domains have the same fraction, the conventional components of the average spin Hall conductivities still exist, while the unconventional components will be cancelled. That deviates from our experimental observation, where the unconventional spin-Hall torques are robust.

We note, however, that a small tetragonal distortion exists in our sample. With the strain from the substrate, the tetragonality or *c/a* ratio for Mn_3GaN is slightly smaller than 1. Such a tensile strain can introduce a small in-plane net magnetic moment along [110] directions[1]. The presence of such an in-plane net moment ensures the control of the magnetic order parameters in the domains[15,16]. We note that the Γ^{5g} representation, which was robustly established by neutron diffraction for the 250 nm samples, does not

admit a FM moment or anomalous Hall effect. The fact that both seem to exist in the thinner 20-nm samples emphasize the possible role of strain in modifying the magnetic and magnetotransport properties of this material.

Supplementary Table 2: The spin Hall conductivity tensors for different domains.

	σ^x	σ^y	σ^z
General tensor	$\begin{bmatrix} \sigma^x_{xx} & \sigma^x_{xy} & \sigma^x_{xz} \\ \sigma^x_{yx} & \sigma^x_{yy} & \sigma^x_{yz} \\ \sigma^x_{zx} & \sigma^x_{zy} & \sigma^x_{zz} \end{bmatrix}$	$\begin{bmatrix} \sigma^y_{xx} & \sigma^y_{xy} & \sigma^y_{xz} \\ \sigma^y_{yx} & \sigma^y_{yy} & \sigma^y_{yz} \\ \sigma^y_{zx} & \sigma^y_{zy} & \sigma^y_{zz} \end{bmatrix}$	$\begin{bmatrix} \sigma^z_{xx} & \sigma^z_{xy} & \sigma^z_{xz} \\ \sigma^z_{yx} & \sigma^z_{yy} & \sigma^z_{yz} \\ \sigma^z_{zx} & \sigma^z_{zy} & \sigma^z_{zz} \end{bmatrix}$
D1	$\begin{bmatrix} 0 & -a & a \\ b & -c & -d \\ -b & d & c \end{bmatrix}$	$\begin{bmatrix} c & -b & d \\ a & 0 & -a \\ -d & b & -c \end{bmatrix}$	$\begin{bmatrix} -c & -d & b \\ d & c & -b \\ -a & a & 0 \end{bmatrix}$
D2	$\begin{bmatrix} 0 & a & -a \\ -b & -c & -d \\ b & d & c \end{bmatrix}$	$\begin{bmatrix} -c & -b & d \\ a & 0 & a \\ -d & -b & c \end{bmatrix}$	$\begin{bmatrix} c & -d & b \\ d & -c & b \\ a & -a & 0 \end{bmatrix}$
D3	$\begin{bmatrix} 0 & a & a \\ -b & c & -d \\ -b & d & -c \end{bmatrix}$	$\begin{bmatrix} -c & b & d \\ -a & 0 & -a \\ -d & b & c \end{bmatrix}$	$\begin{bmatrix} -c & -d & -b \\ d & c & b \\ a & -a & 0 \end{bmatrix}$
D4	$\begin{bmatrix} 0 & -a & -a \\ b & -c & -d \\ b & d & -c \end{bmatrix}$	$\begin{bmatrix} c & b & d \\ -a & 0 & a \\ -d & -b & -c \end{bmatrix}$	$\begin{bmatrix} c & -d & -b \\ d & -c & -b \\ a & a & 0 \end{bmatrix}$
Average	$\begin{bmatrix} 0 & 0 & 0 \\ 0 & 0 & -d \\ 0 & d & 0 \end{bmatrix}$	$\begin{bmatrix} 0 & 0 & d \\ 0 & 0 & 0 \\ -d & 0 & 0 \end{bmatrix}$	$\begin{bmatrix} 0 & -d & 0 \\ d & 0 & 0 \\ 0 & 0 & 0 \end{bmatrix}$

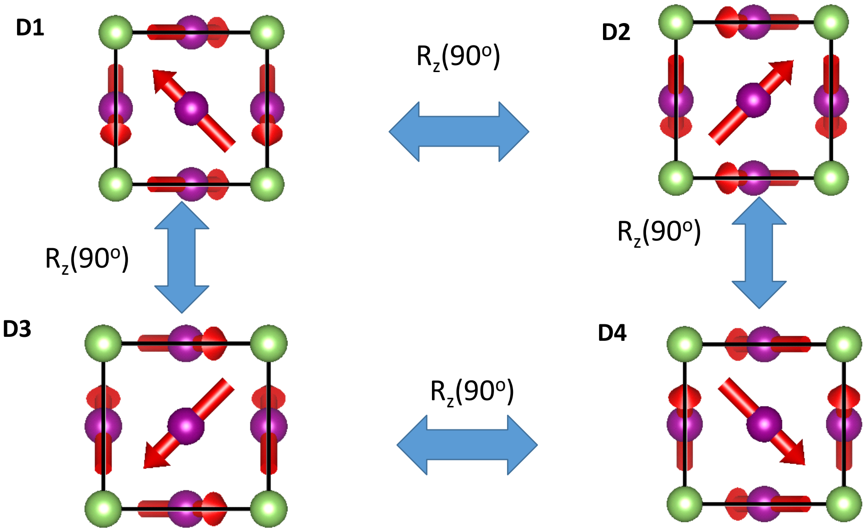

Supplementary Figure 11. Four domains of Mn_3GaN generated by a four-fold rotation around *z* direction.

Supplementary Note 12: Synchrotron spectroscopy and microscopy on Mn_3GaN films

In order to probe the antiferromagnetic domain structure we performed a combination of soft X-ray absorption spectroscopy and microscopy. Figure S12 depicts room temperature X-ray absorption spectroscopy (XAS), X-ray magnetic circular dichroism (XMCD) and X-ray magnetic linear dichroism (XMLD) measurements on a 4 nm Py/ 35 nm Mn_3GaN sample on the LSAT substrate (without a Cu spacer). XMCD spectroscopy measurements, in a grazing incidence geometry with a 0.3 T magnetic field applied along the [110] direction, indicate that a net ferromagnetic moment is present in both the permalloy (green curve in Supplementary Figure 12a) and Mn_3GaN (blue curve in Supplementary Figure 12b) layers.

X-ray magnetic linear dichroism (XMLD) at the Mn $L_{2,3}$ edge was measured in a normal-incidence x-ray geometry with magnetic field held along one of the [110] type in-plane directions and x-ray polarization axis projected along both orthogonal [110] type axes. The non-zero XMLD spectral intensity confirms that the population of canted antiferromagnetic domains can be influenced by the rotation of the Py layer and is not simply a random and equal distribution of domain variants as elaborated on in section XI. We note that if the antiferromagnetic domain population were determined by the bulk domain variant degeneracy, the linear dichroism projections averaged across the beam area (approximately 150 x 500 microns) would cancel each other out and no net XMLD intensity would be measured. Furthermore, no evidence of oxidation was seen at the Fe (black curve left panel) or Mn (black curve middle panel) $L_{2,3}$ edge XAS lineshape. Hence the XAS data indicate that metal oxide antiferromagnetic phases are not present and that the XMLD signal hence originates from the Mn_3GaN layer.

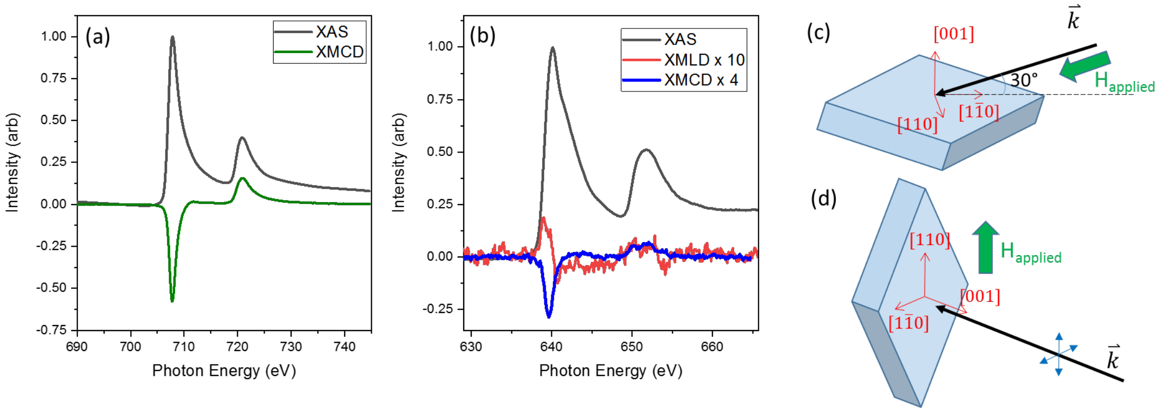

Supplementary Figure 12. Room temperature magnetic spectroscopy on MGN/Permalloy bilayer at the (a) Fe and (b) Mn L edges. Field-dependent XMLD indicates an unequal population of antiferromagnetic domains, and XMCD measurements indicate that net ferromagnetic moments are present at the Mn edge. The measurement geometry was (c) grazing incidence for the PEEM and XMCD spectroscopy measurements and normal incidence for the XMLD data. A 0.3 T magnetic field was applied along the beam direction for XMCD spectroscopy and slightly canted towards [001] from the [110] direction for XMLD spectroscopy.

To further probe the non-equal domain population, spatially resolved XMLD-PEEM and XMCD-PEEM mappings of a single layer Mn_3GaN sample (without the Py capping) were taken in zero magnetic field at the peak energies in dichroism (from Supplementary Figure 12). Supplementary Figure 13 shows the XMLD-PEEM and XMCD-PEEM images obtained with the x-ray incidence direction along an in-plane [110] direction of the Mn_3GaN, revealing micron-sized XMLD domains and sub-micron sized XMCD domain regions at room temperature. There is a correlation between the contrast of the XMLD and XMCD domain locations; within each bright XMLD region there is strong contrast between XMCD domains, while

in dark XMLD regions, there is much weaker XMCD contrast. This suggests that XMCD can be used to identify sub-regions of the XMLD regions, with the brightest XMCD domains having a small net moment along $[1\bar{1}0]$ and darkest XMCD domains having a net moment along $[\bar{1}10]$ according to the schematic in Supplementary Figure 13(c). The characteristic length scale of these canted antiferromagnetic domains is on the order of 200-300 nm. We note that the correlation between XMCD and XMLD domains observed here is consistent with the possible antiferromagnetic domain variants as described in Supplementary Figure 11.

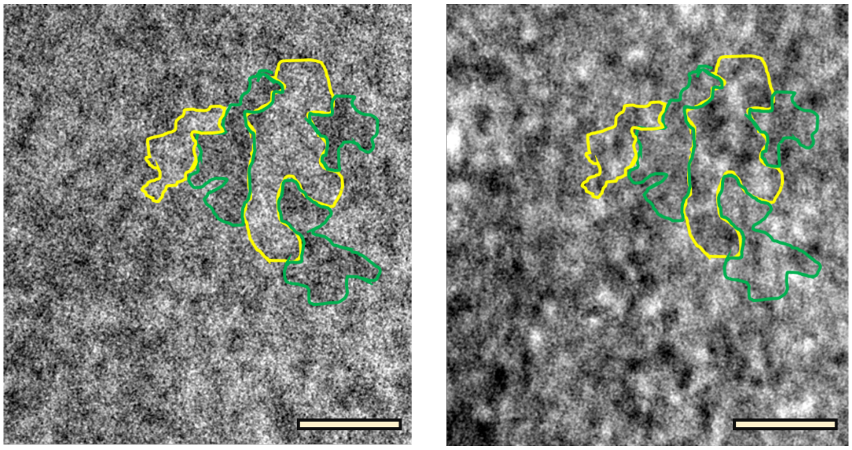

Supplementary Figure 13. Comparison of Mn L edge XMLD (left) and XMCD (right) images at room temperature for a single layer Mn_3GaN film in the same region of the film. The x-rays are incident along the in-plane [110] direction and the scale bars are 1 micron.

To verify that the XMCD domain contrast is due to the frustrated antiferromagnetic order in Mn_3GaN, the sample was heated to above the Néel temperature, and then cooled to room temperature and imaged in the same region. Supplementary Figure 14 illustrates that the XMCD domain contrast vanishes at 360 K, and a different pattern of XMCD domains emerges after the sample is cooled to room temperature, albeit at a lower contrast level. This shows that the XMCD contrast is not localized to specific structural defects at the surface of the film but behaves similarly to other ferromagnetic and antiferromagnetic systems when cycled above their transition temperature. Moreover, radial autocorrelation analysis of the PEEM data (Supplementary Figure 15) gives a similar domain structure after thermal cycling to 360 K, revealing a significant spectral weight out to 200 nm and a pronounced shoulder at 100 nm for both 295 K images, illustrating the consistent length scale of the re-emergent domains seen in the real-space images.

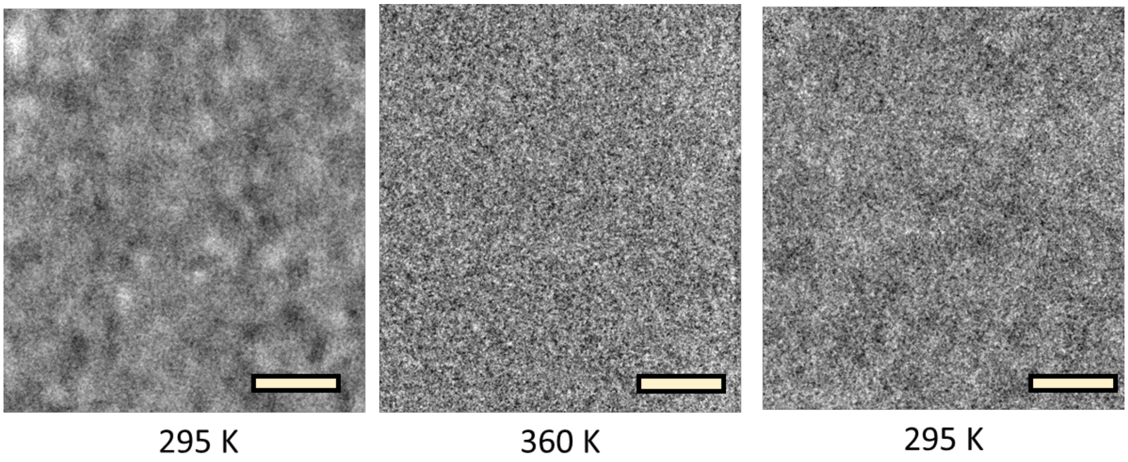

Supplementary Figure 14. Mn L-edge XMCD images as a function of temperature for a single layer Mn_3GaN film taken in the same region of the sample with the same field of view. The x-rays are incident along the in-plane $[\bar{1}10]$ direction and the scale bars are 1 micron. Contrast levels set to the same range (+/- 0.5% XMCD asymmetry) for all images. Domain contrast disappears by 360 K (center), and randomized domains re-emerge when the sample is cooled back down to room temperature (right).

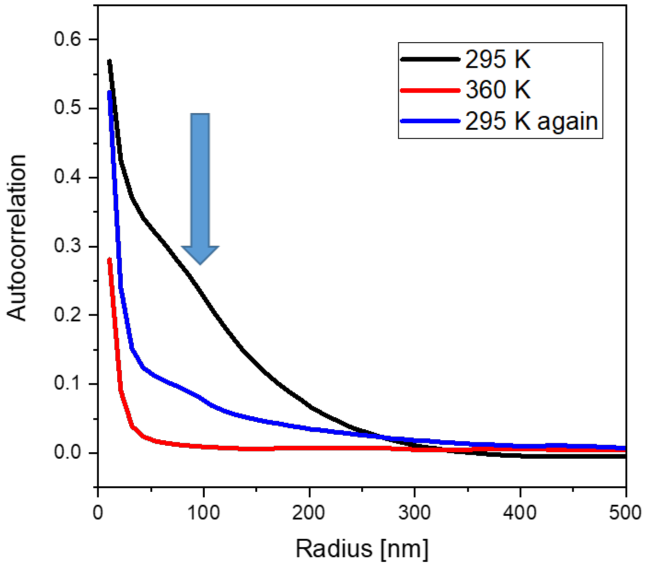

Figure S15. Radial autocorrelation of XMLD images before and after heating sample to 360 K. The arrow points at the pronounced shoulder at 100 nm for both 295 K images.

1. Lukashev, P., Sabirianov, R. F. & Belashchenko, K. Theory of the piezomagnetic effect in Mn-based antiperovskites. *Phys. Rev. B* **78**, 184414 (2008).
2. Kasugai, D., Ozawa, A., Inagaki, T. & Takenaka, K. Effects of nitrogen deficiency on the magnetostructural properties of antiperovskite manganese nitrides. *J. Appl. Phys.* **111**, 07E314 (2012).
3. Zemen, J. *et al.* Frustrated magnetism and caloric effects in Mn-based antiperovskite nitrides: Ab

initio theory. *Phys. Rev. B* **95**, 184438 (2017).

4. Rodríguez-Carvajal, J. Recent advances in magnetic structure determination by neutron powder diffraction. *Phys. B Condens. Matter* **192**, 55–69 (1993).

5. Campbell, B. J., Stokes, H. T., Tanner, D. E. & Hatch, D. M. ISODISPLACE: A web-based tool for exploring structural distortions. *J. Appl. Crystallogr.* **39**, 607–617 (2006).

6. H. T. Stokes, D. M. Hatch, and B. J. C. Isotropy suite. (2007).

7. Slonczewski, J. C. Current-driven excitation of magnetic multilayers. *J. Magn. Magn. Mater.* **159**, L1–L7 (1996).

8. Tshitoyan, V. *et al.* Electrical manipulation of ferromagnetic NiFe by antiferromagnetic IrMn. *Phys. Rev. B* **92**, 214406 (2015).

9. Zhang, W. *et al.* All-electrical manipulation of magnetization dynamics in a ferromagnet by antiferromagnets with anisotropic spin Hall effects. *Phys. Rev. B* **92**, 144405 (2015).

10. Seemann, M., Ködderitzsch, D., Wimmer, S. & Ebert, H. Symmetry-imposed shape of linear response tensors. *Phys. Rev. B* **92**, 155138 (2015).

11. Železný, J., Zhang, Y., Felser, C. & Yan, B. Spin-Polarized Current in Noncollinear Antiferromagnets. *Phys. Rev. Lett.* **119**, 187204 (2017).

12. Zhang, Y., Železný, J., Sun, Y., Van Den Brink, J. & Yan, B. Spin Hall effect emerging from a noncollinear magnetic lattice without spin-orbit coupling. *New J. Phys.* **20**, 073028 (2018).

13. J. Železný. Linear response symmetry. https://bitbucket.org/zeleznyj/linear-response-symmetry.

14. Zhang, W. *et al.* Giant facet-dependent spin-orbit torque and spin Hall conductivity in the triangular antiferromagnet IrMn3. *Sci. Adv.* **2**, e1600759 (2016).

15. Nakatsuji, S., Kiyohara, N. & Higo, T. Large anomalous Hall effect in a non-collinear antiferromagnet at room temperature. *Nature* **527**, 212–215 (2015).

16. Higo, T. *et al.* Large magneto-optical Kerr effect and imaging of magnetic octupole domains in an antiferromagnetic metal. *Nat. Photonics* **12**, 73–78 (2018).
